# Partial Differential Equation-Constrained Diffeomorphic Registration from Sum of Squared Differences to Normalized Cross-Correlation, Normalized Gradient Fields, and Mutual Information: A Unifying Framework

**DOI:** 10.3390/s22103735

**Published:** 2022-05-13

**Authors:** Monica Hernandez, Ubaldo Ramon-Julvez, Daniel Sierra-Tome

**Affiliations:** 1 Aragon Institute of Engineering Research (I3A), 50018 Zaragoza, Spain; 2 Department of Computer Sciences, University of Zaragoza (UZ), 50018 Zaragoza, Spain; ubi1616@gmail.com (U.R.-J.); sierradaniel.ind@gmail.com (D.S.-T.)

**Keywords:** PDE-LDDMM, Gauss–Newton–Krylov, optimal control optimization, band-limited vector fields, normalized cross-correlation, normalized gradient fields, mutual information

## Abstract

This work proposes a unifying framework for extending PDE-constrained Large Deformation Diffeomorphic Metric Mapping (PDE-LDDMM) with the sum of squared differences (SSD) to PDE-LDDMM with different image similarity metrics. We focused on the two best-performing variants of PDE-LDDMM with the spatial and band-limited parameterizations of diffeomorphisms. We derived the equations for gradient-descent and Gauss–Newton–Krylov (GNK) optimization with Normalized Cross-Correlation (NCC), its local version (lNCC), Normalized Gradient Fields (NGFs), and Mutual Information (MI). PDE-LDDMM with GNK was successfully implemented for NCC and lNCC, substantially improving the registration results of SSD. For these metrics, GNK optimization outperformed gradient-descent. However, for NGFs, GNK optimization was not able to overpass the performance of gradient-descent. For MI, GNK optimization involved the product of huge dense matrices, requesting an unaffordable memory load. The extensive evaluation reported the band-limited version of PDE-LDDMM based on the deformation state equation with NCC and lNCC image similarities among the best performing PDE-LDDMM methods. In comparison with benchmark deep learning-based methods, our proposal reached or surpassed the accuracy of the best-performing models. In NIREP16, several configurations of PDE-LDDMM outperformed ANTS-lNCC, the best benchmark method. Although NGFs and MI usually underperformed the other metrics in our evaluation, these metrics showed potentially competitive results in a multimodal deformable experiment. We believe that our proposed image similarity extension over PDE-LDDMM will promote the use of physically meaningful diffeomorphisms in a wide variety of clinical applications depending on deformable image registration.

## 1. Introduction

In the past two decades, diffeomorphic registration has become a fundamental problem in medical image analysis [1]. The diffeomorphic transformations estimated from the solution of the image registration problem constitute the inception point in Computational Anatomy studies for modeling and understanding population trends and longitudinal variations, and for establishing relationships between imaging phenotypes and genotypes in Imaging Genetics [2,3,4,5,6,7,8]. Moreover, diffeomorphic registration can be as useful as any other deformable image registration framework in the fusion of multi-modal information from different sensors, the capture of correlations between structure and function, the guidance of computerized interventions, and many other applications [9,10,11].

A relevant issue in deformable image registration is the quest for the most sensible transformation model for each clinical domain. On the one hand, there are domains where the underlying biophysical model of the transformation is known. The incompressible motion of the healthy heart is a relevant example [12]. On the other hand, there are also important clinical contexts where the deformation model is not known, although there is active research on finding the most plausible transformation among those explained by a physical model [13,14,15]. The most relevant examples are the deformation between healthy and diseased brains or the longitudinal evolution of the brain changes in healthy and diseased individuals.

Although the differentiability and invertibility of the diffeomorphisms constitute fundamental features for Computational Anatomy, the diffeomorphic constraint does not necessarily guarantee that a transformation computed with a given method is physically meaningful for the clinical domain of interest. In order to obtain physically meaningful diffeomorphisms, the diffeomorphic registration methods should be able to impose a plausible physical model to the computed transformations.

PDE-constrained LDDMM (PDE-LDDMM) registration methods have arisen as an appealing paradigm for computing diffeomorphisms under plausible physical models [13,14,15,16,17,18,19,20,21,22]. In the cases where the model is known, the PDE-LDDMM formulation allows the introduction of the priors of the particular model [23,24,25,26]. The PDE-constrained formulation is also helpful in the quest of plausible transformations for a clinical application with an unknown deformation model [15]. In addition, PDE-LDDMM is a well-suited approach for the estimation of registration uncertainty [27,28].

The different PDE-LDDMM methods differ on the variational problem formulation, diffeomorphism parameterization, regularizers, image similarity metrics, optimization methods, and additional PDE constraints. From them, the use of Gauss–Newton–Krylov optimization [13,14,22], the addition of nearly incompressible terms in the variational formulation [14,19], the use of variants involving the deformation state equation [18,22], and the introduction of the band-limited parameterization and HPC or GPU implementations [19,20,22,29], constitute the most successful contributions to the realistic and efficient computation of physically meaningful diffeomorphisms so far. Some attention has been given to the image similarity metric, where the sum of squared differences (SSD) between the final state variable and the target image has been mostly used [13,16,22]. Only two variations of PDE-LDDMM with normalized gradient fields (NGFs) and mutual information (MI) have been proposed in [18,30] (*ArXiv* paper). These two methods use gradient-based techniques for optimization.

SSD is based on image subtraction, so it is only well suited in uni-modal registration for images where the intensity of the reciprocal structures do not vary much. Indeed, SSD is not robust to noise, intensity inhomogeneity, and partial volume effects. Moreover, SSD is not suitable for multi-modal registration, even for transformation models with a few degrees of freedom such as those in rigid or affine image registration [31,32]. Therefore, there is a need for PDE-LDDMM methods, preferably with Gauss–Newton–Krylov optimization, which, apart from SSD, can support alternative image similarity metrics better behaved than SSD. The solution to this problem will promote the use of PDE-LDDMM in a wide variety of clinical applications depending on deformable image registration where images are acquired from different sensors.

This work proposes a unifying framework for introducing different image similarity metrics in the two best-performing variants of PDE-LDDMM [22,33]. From the Lagrangian variational problems, we have identified that a change in the image similarity metric involves changing the initial adjoint and the initial incremental adjoint variables. We have derived the equations of these variables needed for gradient-descent and Gauss–Newton–Krylov optimization with Normalized Cross Correlation (NCC), its local version (lNCC), Normalized Gradient Fields (NGFs), and Mutual Information (MI).

NCC, lNCC, and MI have accompanied SSD in different deformable image registration methods since their inception [34]. NGFs is an interesting metric for a wide variety of deformable registration problems [35]. These metrics are available in relevant deformable image registration packages such as the Insight Toolk it (www.itk.org, accessed on 1 January 2022), NiftyReg (https://sourceforge.net/projects/niftyreg, accessed on 1 January 2022), or Fair [32], among others. In the framework of diffeomorphic image registration, ANTS registration was implemented for SSD, lNCC, and MI (www.nitrc.org/projects/ants, accessed on 1 January 2022) and the performance of the different image similarity metrics was evaluated in [36]. We have selected these metrics as a starting point, although our framework is extensible to other image similarity metrics proposed in the literature, provided that the first and second-order variations of the image similarity metric can be written in the expected form [37,38].

Our experiments focused on the spatial (SP) and band-limited (BL) stationary parameterization of diffeomorphisms, although the proposed methods can be straightforwardly extended to the non-stationary parameterization [39]. We have obtained successful Gauss–Newton–Krylov methods for SSD, NCC, and lNCC with evaluation results greatly overpassing gradient-descent and competing with the respective version of ANTS diffeomorphic registration [40]. For NGFs, the second-order method did not provide satisfactory results in comparison with gradient-descent. For MI, the memory load of the second-order method hindered a proper evaluation in 3D datasets. We extensively studied the performance of our methods in NIREP16 and Klein et al.’s evaluation frameworks [41,42], obtaining an interesting insight into the impact of the different image similarity metrics in the PDE-LDDMM framework.

Since the advances that made it possible to learn the optical flow using convolutional neural networks (FlowNet [43]), dozens of deep-learning data-based methods were proposed to approach the problem of deformable image registration in different clinical applications [44]. Some of them are specifically devised for diffeomorphic registration where the different LDDMM ingredients are used as a backbone for diffeomorphism parameterization and the definition of the loss functions [45,46,47,48,49,50,51,52,53,54]. These methods use SSD and NCC metrics in the image similarity loss function, and the proposed models are usually limited to a single modality where the appearance of the image pairs needs to be similar to the training data. From them, only SynthMorph proposed a model valid for multi-modal registration through the extensive generation of simulated data for training, which yields a fast inference for diffeomorphism computation once the difficulties with training have been overcome [53].

Although the authors of SynthMorph provided an extensive study on the generalization capability of their models in different multi-modal experiments, the loss function is restricted to the Dice Similarity Coefficient (DSC) on image segmentations and, therefore, interesting questions such as the actual ability of deep-learning models to deal with multimodality or influence of the image similarity loss function on the registration accuracy have not been answered. In addition, results from the Learn2Reg challenge question the superiority of deep learning approaches and open new research directions into hybrid methods for which contributions to traditional optimization-based methods like ours may be of interest [55].

In the following, Section 2 reviews the foundations of PDE-LDDMM and BL PDE-LDDMM, from the original variant proposed in [13,16] to the variants used in this work. Section 4.5 analyzes the change of image similarity metrics in PDE-LDDMM and derives the equations needed for gradient-descent and Gauss–Newton–Krylov optimization for the considered metrics. Section 4 gathers the experimental setup for the evaluation of the methods, the numerical and implementation details of the proposed methods and the benchmarks. Section 5 shows the evaluation results. Finally, Section 7 gathers the most remarkable conclusions of our work.

## 2. PDE-Constrained LDDMM

### 2.1. LDDMM

LDDMM was proposed by Beg et al. in [56]. In this section, we recall the most relevant aspects of this interesting method, which underpin PDE-LDDMM. Let $\Omega \in R^{d}$, *d* = 2, 3 be the image domain. The LDDMM registration problem is formulated between the source and the target images, $I_{0}:\Omega \to R$ and $I_{1}:\Omega \to R$. These images are square-integrable functions on Ω. Diff(Ω) represents the Riemannian manifold of smooth diffeomorphisms on Ω. The tangent space at the identity diffeomorphism is denoted with *V*. The Riemannian metric of Diff(Ω) is defined from a scalar product in *V*

(1) $${\langle v,w\rangle }_{V}={\langle Lv,w\rangle }_{L^{2}}=\int_{\Omega }\langle Lv\left(x\right),w\left(x\right)\rangle d\Omega ,$$

through the invertible self-adjoint operator $L={\left(Id-\alpha \Delta \right)}^{s},\alpha >0,s\in N$ with inverse *K*.

LDDMM aims at finding a smooth map $\varphi :\Omega \to R^{d}$ with smooth inverse such that the warped initial image $I_{0}\circ \varphi$ is non-rigidly aligned with $I_{1}$. The diffeomorphism φ is parameterized in the tangent space *V* from a time-varying vector field flow $v:\Omega \times \left[0,1\right]\to R^{d}$ and a path in Diff(Ω) $\phi :\Omega \times \left[0,1\right]\to R^{d}$ such that *v* and ϕ satisfy the PDE

(2) $$\partial_{t}\phi \left(t\right)+D\phi \left(t\right)\cdot v_{t}=0$$

with initial condition ϕ(0)=id. It holds that φ=ϕ(1).

The solution to the registration problem is obtained from the minimization of a variational problem

(3) $$E\left(v\right)=\frac{1}{2}E_{reg}\left(v\right)+\frac{1}{\sigma^{2}}E_{img}\left(I_{0},I_{1},\varphi \right),$$

where $E_{reg}\left(v\right)$ is the regularization term, $E_{img}\left(I_{0},I_{1},\varphi \right)$ is the image similarity metric that quantifies the differences between $I_{0}\circ \varphi$ and $I_{1}$ after registration, and σ weights the contribution of both terms to the total energy.

### 2.2. Original PDE-Constrained LDDMM

PDE-constrained LDDMM (PDE-LDDMM) was originally formulated as a constrained variational problem from the minimization of

(4) $$E\left(v\right)=\frac{1}{2}\int_{0}^{1}{\langle v_{t},v_{t}\rangle }_{V}dt+\frac{1}{\sigma^{2}}{∥m\left(1\right)-I_{1}∥}_{L^{2}}^{2},$$

subject to

(5) $$\partial_{t}m\left(t\right)+\nabla m\left(t\right)\cdot v_{t}=0,$$

with initial condition $m\left(0\right)=I_{0}$ [14,16]. The solution of Equation (5), m(1), is the warped initial image. The image similarity metric is the sum of squared differences (SSD) between the intensities of m(1) and $I_{1}$.

Although in the great majority of LDDMM methods the optimization is approached with gradient-descent [16,17,18,40,56,57,58], Gauss–Newton–Krylov optimization has emerged as the method of choice for PDE-LDDMM due to the excellent numerical accuracy and the extraordinarily fast convergence rate [13,14,19]. The first and second-order differentials of the PDE-constrained variational problem are computed using the method of Lagrange multipliers, as follows.

Let us define the Lagrange multipliers λ:Ω×[0,1]→R and η:Ω→R associated with Equation (5) and its initial condition. The Lagrangian functional corresponds to the expression

(6) $$E_{Lag}\left(v\right)=E\left(v\right)+\int_{0}^{1}{\langle \lambda \left(t\right),\partial_{t}m\left(t\right)+Dm\left(t\right)\cdot v_{t}\rangle }_{L^{2}}dt+{\langle \eta ,m\left(0\right)-I_{0}\rangle }_{L^{2}}.$$

The first-order variation of the Lagrangian yields the expression of the gradient

(7) $$\nabla_{v}E_{Lag}\left(v\right)=Lv+\lambda \cdot \nabla m,$$

where   

(8) $$\begin{array}{c} \partial_{t}m\left(t\right)+\nabla m\left(t\right)\cdot v_{t}=0 \end{array}$$

(9) $$\begin{array}{c} -\partial_{t}\lambda \left(t\right)-\nabla \cdot \left(\lambda \left(t\right)\cdot v_{t}\right)=0, \end{array}$$

subject to the initial and final conditions $m\left(0\right)=I_{0}$ and $\lambda \left(1\right)=-\frac{2}{\sigma^{2}}\left(m\left(1\right)-I_{1}\right)$. Equation (4) subject to Equation (5) is referred to as an optimal control problem, where *v* is the control variable, Equation (8) is the state equation and Equation (9) is the adjoint equation.

The second-order variation of the Lagrangian functional yields the expression of the Hessian-vector product, written in Gauss–Newton positive-definite approximated form,

(10) $$H_{v}E_{Lag}\left(v\right)\delta v=L\delta v+\delta \lambda \cdot \nabla m,$$

where

(11) $$-\partial_{t}\delta \lambda \left(t\right)-\nabla \cdot \left(\delta \lambda \left(t\right)\cdot v_{t}\right)=0,$$

with final condition $\delta \lambda \left(1\right)=-\frac{2}{\sigma^{2}}\delta m\left(1\right)$. The variation of *m*, δm, satisfies the PDE with initial condition δm(0)=0

(12) $$\partial_{t}\delta m\left(t\right)+\nabla \delta m\cdot v_{t}+\nabla m\cdot \delta v=0.$$

Optimization using gradient-descent in *V* is driven by the update equation

(13) $$v^{n+1}=v^{n}-\epsilon K\nabla_{v}E_{Lag}\left(v\right),$$

while Gauss–Newton–Krylov optimization yields the update equation

(14) $$v^{n+1}=v^{n}+\epsilon \delta v^{n},$$

where $\delta v^{n}$ is computed from preconditioned conjugate gradient (PCG) on the system

(15) $$H_{v}E_{Lag}\left(v^{n}\right)\delta v^{n}=-\nabla_{v}E_{Lag}\left(v^{n}\right),$$

where $H_{v}E_{Lag}\left(v^{n}\right)$ is the positive-definite Hessian approximation. The preconditioner used in this work is *K* [13].

### 2.3. Variants of PDE-LDDMM

The original PDE-LDDMM has been recently completed with two alternative variants [22]. They can be considered theoretically but not numerically equivalent formulations of the original one. These variants have been shown to improve the original formulation in terms of registration accuracy and efficiency, in combination with the use of the band-limited parameterization and GPU implementation [20,22,33].

#### 2.3.1. Variant I

The first variant departs from the original variational formulation (Equation (4)) by replacing the solution of the state equation (Equations (5) and (8)) with the identity

(16) $$m\left(t\right)=I_{0}\circ \phi \left(t\right),$$

where ϕ(t) is computed from the solution of the deformation state equation

(17) $$\partial_{t}\phi \left(t\right)+D\phi \left(t\right)\cdot v_{t}=0$$

with initial condition ϕ(0)=id. Analogously, the solution of the adjoint equation (Equation (9)) is replaced with the identity

(18) $$\lambda \left(t\right)=|D\phi^{-1}\left(t\right)|\lambda \left(1\right)\circ \phi^{-1}\left(t\right),$$

and the solution of the incremental adjoint equation (Equation (11)) is replaced with the identity

(19) $$\delta \lambda \left(t\right)=|D\phi^{-1}\left(t\right)|\delta \lambda \left(1\right)\circ \phi^{-1}\left(t\right)\cdot \delta \phi^{-1}\left(t\right).$$

The inverse diffeomorphism $\psi \left(t\right)=\phi^{-1}\left(t\right)$ and the corresponding Jacobian determinant $J\left(t\right)=|D\phi^{-1}\left(t\right)|$ are computed, respectively, from the inverse deformation state equation and the inverse Jacobian equation

(20) $$\begin{array}{c} -\partial_{t}\psi \left(t\right)-D\psi \left(t\right)\cdot v_{t}=0 \end{array}$$

(21) $$\begin{array}{c} -\partial_{t}J\left(t\right)-v_{t}\cdot \nabla J\left(t\right)=-J\left(t\right)\nabla \cdot v_{t} \end{array}$$

with final conditions ψ(1)=id and J(1)=1. These identities were proposed in [16,22,56] and effectively used in [22].

#### 2.3.2. Variant II

The second variant consists in replacing the state equation (Equation (5)) by the deformation state equation (Equation (17)) in the original variational formulation (Equation (4)) [18,22]. In this case, the Lagrangian corresponds to

(22) $$\begin{array}{c} E_{Lag}\left(v\right)=E\left(v\right)+\int_{0}^{1}\langle \rho \left(t\right),\partial_{t}\phi \left(t\right)+\;\;\;\;\;\;\;\;\;\;\;\;\;\;\;\;\;\\ \;\;\;\;\;\;\;\;\;\;\;\;\;\;\;\;\;D\phi \left(t\right)\cdot v_{t}\rangle_{L^{2}}dt+{\langle \mu ,\phi \left(0\right)-id\rangle }_{L^{2}}, \end{array}$$

where the Lagrange multipliers are $\rho :\Omega \times \left[0,1\right]\to R^{d}$, associated with the deformation state equation, and $\mu :\Omega \to R^{d}$, associated with its initial condition.

For Variant II, the first-order variation of the Lagrangian yields the expression of the gradient

(23) $$\nabla_{v}E_{Lag}\left(v\right)=Lv+D\phi \cdot \rho ,$$

where

(24) $$\begin{array}{c} \partial_{t}\phi \left(t\right)+D\phi \left(t\right)\cdot v_{t}=0 \end{array}$$

(25) $$\begin{array}{c} -\partial_{t}\rho \left(t\right)-\nabla \cdot \left(\rho \left(t\right)\cdot v_{t}\right)=0 \end{array}$$

subject to the initial and final conditions ϕ(0)=id, and ρ(1)=λ(1)·∇m(1).

The second-order variation of the Lagrangian functional yields the expression of the Hessian-vector product (in Gauss–Newton approximated form)

(26) $$H_{v}E_{Lag}\left(v\right)\delta v=L\delta v+D\delta \phi \cdot \rho ,$$

where

(27) $$\begin{array}{c} \partial_{t}\delta \phi \left(t\right)+D\delta \phi \left(t\right)\cdot v_{t}+D\phi \left(t\right)\cdot \delta v\left(t\right)=0 \end{array}$$

(28) $$\begin{array}{c} -\partial_{t}\delta \rho \left(t\right)-\nabla \cdot \left(\delta \rho \left(t\right)\cdot v_{t}\right)=0 \end{array}$$

subject to δϕ(1)=0, δρ(1)=δλ(1)·∇m(1).

### 2.4. Band-Limited PDE-LDDMM

The band-limited (BL) parameterization of diffeomorphisms was proposed by Zhang et al. [58,59,60]. In this section, we recall the most relevant aspects of this parameterization and then describe BL PDE-LDDMM. Let $\tilde{\Omega }$ be the discrete Fourier domain truncated with frequency bounds $K_{1},$⋯,$K_{d}$. We denote with $\tilde{V}$ the space of discretized band-limited vector fields on Ω with these frequency bounds. The elements in $\tilde{V}$ are represented in the Fourier domain as $\tilde{v}:\tilde{\Omega }\to C^{d}$, $\tilde{v}\left(k_{1},\cdots ,k_{d}\right)$, and in the spatial domain as $\iota \left(\tilde{v}\right):\Omega \to R^{d}$, $\iota \left(\tilde{v}\right)\left(x_{1},\cdots ,x_{d}\right)$. The application $\iota :\tilde{V}\to V$ denotes the natural inclusion mapping of $\tilde{V}$ in *V*. The application $\pi :V\to \tilde{V}$ denotes the projection of *V* onto $\tilde{V}$.

We denote with $Diff(\tilde{\Omega })$ the finite-dimensional Riemannian manifold of diffeomorphisms on $\tilde{\Omega }$ with corresponding Lie algebra $\tilde{V}$. The Riemannian metric in $Diff(\tilde{\Omega })$ is defined from the scalar product

(29) $${\langle \tilde{v},\tilde{w}\rangle }_{\tilde{V}}={\langle \tilde{L}\tilde{v},\tilde{w}\rangle }_{l^{2}},$$

where $\tilde{L}$ is the projection of operator *L* in the truncated Fourier domain. Similarly, we will denote with $\tilde{\Upsilon }$ the projection of any linear operator Υ in the truncated Fourier domain. In addition, we will denote with ★ the truncated convolution.

The BL PDE-LDDMM SSD variational problem is given by the minimization of

(30) $$E\left(\tilde{v}\right)=\frac{1}{2}\int_{0}^{1}{\langle \tilde{L}{\tilde{v}}_{t},{\tilde{v}}_{t}\rangle }_{l^{2}}dt+\frac{1}{\sigma^{2}}{∥m\left(1\right)-I_{1}∥}_{L^{2}}^{2}$$

subject to the state equation

(31) $$\partial_{t}m\left(t\right)+\nabla m\left(t\right)\cdot \iota \left({\tilde{v}}_{t}\right)=0$$

with initial condition $m\left(0\right)=I_{0}$.

The expression of the gradient is computed in the space of band-limited vectors yielding

(32) $$\overset{˜}{\nabla_{\tilde{v}}E_{Lag}\left(\tilde{v}\right)}=\tilde{L}\tilde{v}+\pi \left(\lambda \cdot \nabla m\right).$$

The expression of the Hessian-vector product is computed analogously, yielding

(33) $$\overset{˜}{H_{\tilde{v}}E_{Lag}\left(\tilde{v}\right)}\delta \tilde{v}=\tilde{L}\delta \tilde{v}+\pi \left(\delta \lambda \cdot \nabla m\right).$$

Optimization using gradient-descent is driven by the update equation

(34) $${\tilde{v}}^{n+1}={\tilde{v}}^{n}-\epsilon \tilde{K}\overset{˜}{\nabla_{\tilde{v}}E_{Lag}\left(\tilde{v}\right)},$$

while Gauss–Newton–Krylov optimization yields the update equation

(35) $${\tilde{v}}^{n+1}={\tilde{v}}^{n}+\epsilon \delta {\tilde{v}}^{n},$$

where $\delta {\tilde{v}}^{n}$ is computed from preconditioned conjugate gradient (PCG) on the Hessian-gradient system defined in the BL domain.

#### 2.4.1. BL Variant I

The BL version of Variant I is obtained with the identity $m\left(t\right)=I_{0}\circ \phi \left(t\right)$, where $\phi \left(t\right)=id-\iota (\tilde{u}\left(t\right))$ and $\tilde{u}\left(t\right)$ is the solution of the BL deformation state equation

(36) $$\partial_{t}\tilde{u}\left(t\right)+\tilde{D}\tilde{u}\left(t\right)★{\tilde{v}}_{t}={\tilde{v}}_{t}$$

with initial condition $\tilde{u}\left(0\right)=\pi \left(0_{V}\right)$. Analogously, λ(t) and δλ(t) are obtained from Equations (18) and (19), where the inverse diffeomorphism and its Jacobian are computed from the inverse deformation state equation and the inverse Jacobian equation defined in the space of BL vector fields. The details can be found in [22].

#### 2.4.2. BL Variant II

The BL version of Variant II is given by the minimization of Equation (30) subject to the BL deformation state equation (Equation (36)). The gradient and the Hessian of the Lagrangian are given by

(37) $$\overset{˜}{\nabla_{\tilde{v}}E_{Lag}\left(\tilde{v}\right)}=\tilde{L}\tilde{v}+\tilde{\rho }-\tilde{D}\tilde{u}★\tilde{\rho }$$

(38) $$\overset{˜}{H_{\tilde{v}}E_{Lag}\left(\tilde{v}\right)}\delta \tilde{v}=\tilde{L}\delta \tilde{v}+\delta \tilde{\rho }-\tilde{D}\delta \tilde{u}★\tilde{\rho }.$$

The PDE equations involved in the computation of the gradient and Hessian are the convenient definitions of the spatial PDE equations in the band-limited domain. The specific details can be found in [22]. From them, the most relevant ones to recall are $\tilde{\rho }\left(1\right)=\pi \left(\lambda \left(1\right)\cdot \nabla m\left(1\right)\right)$ and $\delta \tilde{\rho }\left(1\right)=\pi \left(\delta \lambda \left(1\right)\cdot \nabla m\left(1\right)\right)$.

## 3. Extending PDE-LDDMM from SSD to NCC, lNCC, NGFs, and MI Image Similarity Metrics

### 3.1. Changing the Image Similarity Metric in PDE-LDDMM

From the analysis of the equations involved in the computation of Variants I and II with SSD, a change in the image similarity term for PDE-LDDMM supposes to recompute the expressions of λ(1) and δλ(1) for the given metric. This is valid also for Variant II since the adjoint variable ρ(1) depends on λ(1). The BL versions of Variants I and II do also depend on λ(1) and δλ(1).

Following the ideas in [32], our image similarity terms of interest can be written in the shape

(39) $$E_{img}\left(v\right)=\Psi \left(r\left(m\left(1\right)\right)\right),$$

where the dependence of the right-hand-side on *v* is obtained through the state equation. The first-order differential of $E_{img}$ corresponds to

(40) $$\delta E_{img}\left(v\right)=\langle \frac{\partial \Psi }{\partial r}\frac{\partial r}{\partial m(1)},dm\left(1\right)\rangle .$$

Departing from the expression of Equation (A2) in [22], the first-order differential of the Lagrangian functional for a generic image similarity term is given by

(41) $$\begin{array}{c} \delta E_{Lag}=\int_{0}^{1}<Lv,dv>dt+\langle \frac{\partial \Psi }{\partial r}\frac{\partial r}{\partial m(1)},dm\left(1\right)\rangle +\;\;\;\;\\ \;\;\;\;\;\;+\int_{0}^{1}\langle d\lambda ,\partial_{t}m+Dm\cdot v\rangle +\langle \lambda ,\partial_{t}dm+Ddm\cdot v+Dm\cdot dv\rangle +\;\;\\ \;\;\;\;\;\;\;\;\;+\langle d\eta ,m\left(0\right)-I_{0}\rangle +\langle \eta ,dm\left(0\right)\rangle . \end{array}$$

Then, integration by parts combined with the Green formula yield

(42) $$\delta E_{Lag}\left(v\right)=\langle \frac{\partial \Psi }{\partial r}\frac{\partial r}{\partial m(1)}+\lambda \left(1\right),dm\left(1\right)\rangle +other\;terms.$$

The full expression can be found in Equation (A3) in [22]. Since, in particular, $\delta E_{Lag}$ needs to vanish for all dm we have

(43) $$\lambda \left(1\right)=-\frac{\partial \Psi }{\partial r}\frac{\partial r}{\partial m(1)}.$$

Accordingly,

(44) $$\delta^{2}E_{img}\left(v\right)=\langle \langle \langle \frac{\partial r}{\partial m(1)},dm\left(1\right)\rangle ,\frac{\partial^{2}\Psi }{\partial r^{2}}\frac{\partial r}{\partial m(1)}\rangle +\langle dm\left(1\right),\frac{\partial \Psi }{\partial r}\frac{\partial^{2}r}{\partial m{\left(1\right)}^{2}}\rangle ,dm\left(1\right)\rangle .$$

and

(45) $$\begin{array}{c} \delta^{2}E_{img}\left(v\right)=\langle \langle \langle \frac{\partial r}{\partial m(1)},dm\left(1\right)\rangle ,\frac{\partial^{2}\Psi }{\partial r^{2}}\frac{\partial r}{\partial m(1)}\rangle \;\;\;\;\;\;\;\;\;\\ \;\;\;\;\;\;\;\;+\langle dm\left(1\right),\frac{\partial \Psi }{\partial r}\frac{\partial^{2}r}{\partial m{\left(1\right)}^{2}}\rangle +\delta \lambda \left(1\right),dm\left(1\right)\rangle +other\;terms. \end{array}$$

Since $\delta^{2}E_{Lag}$ also needs to vanish for all dm, we have

(46) $$\begin{array}{c} \delta \lambda \left(1\right)=-\langle \frac{\partial r}{\partial m(1)}dm\left(1\right),\frac{\partial^{2}\Psi }{\partial r^{2}}\frac{\partial r}{\partial m(1)}\rangle -\langle dm\left(1\right),\frac{\partial \Psi }{\partial r}\frac{\partial r^{2}}{\partial m{\left(1\right)}^{2}}\rangle . \end{array}$$

For Gauss–Newton–Krylov optimization, δλ(1) is approximated by the positive definite expression

(47) $$\begin{array}{c} \delta \lambda \left(1\right)\approx -\langle \frac{\partial r}{\partial m(1)}dm\left(1\right),\frac{\partial^{2}\Psi }{\partial r^{2}}\frac{\partial r}{\partial m(1)}\rangle , \end{array}$$

that neglects the higher-order derivatives of the inner function *r* [32].

In practise, we compute $\delta E_{img}\left(v\right)$ and $\delta^{2}E_{img}\left(v\right)$ in the form of Equations (40) and (44) and identify λ(1) and δλ(1) within the scalar products

(48) $$\begin{array}{c} \delta E_{img}\left(v\right)=\langle \nabla E_{img}\left(v\right),dm\left(1\right)\rangle \end{array}$$

(49) $$\begin{array}{c} \delta^{2}E_{img}\left(v\right)=\langle \langle dm\left(1\right),HE_{img}\left(v\right)\rangle ,dm\left(1\right)\rangle . \end{array}$$

Alternatively, for some metrics, the expression of δλ(1) can be computed more straightforwardly from the differential of λ(1). The obtained expressions are corroborated by the equations of λ(1) and δλ(1) for the SSD PDE-LDDMM problem with $\psi ={\langle \cdot ,\cdot \rangle }_{L^{2}}$ and $r=m\left(1\right)-I_{1}$. In the following, we derive the expressions of λ(1) and δλ(1) for the image similarity metrics considered in this work.

### 3.2. Normalized Cross-Correlation (NCC)

In PDE-LDMMM, the NCC image similarity metric is defined as

(50) $$E_{img}\left(v\right)=\frac{1}{\sigma^{2}}\int_{\Omega }1-\left(\frac{{\langle \bar{m}\left(1\right),{\bar{I}}_{1}\rangle }^{2}}{∥\bar{m}{\left(1\right)∥}^{2}\cdot {∥{\bar{I}}_{1}∥}^{2}}\right)\left(x\right)d\Omega ,$$

where $\bar{I}=I-\mathrm{mean}\left(I\right)$ for a generic image *I*. Let us define $A\left(x\right)=\langle \bar{m}\left(1\right)\left(x\right),{\bar{I}}_{1}\left(x\right)\rangle$, $B\left(x\right)=∥\bar{m}{\left(1\right)\left(x\right)∥}^{2}$, and $C\left(x\right)=∥{\bar{I}}_{1}{\left(x\right)∥}^{2}$. This allows us to define the NCC image similarity metric in the form of

(51) $$E_{img}\left(v\right)=\frac{1}{\sigma^{2}}\int_{\Omega }1-r\left(x\right)d\Omega ,$$

where $r=\frac{A^{2}}{B\cdot C}$.

Using the expression of the differential dr, $dA=\langle {\bar{I}}_{1},d\bar{m}\left(1\right)\rangle$, and $dB=2\langle \bar{m}\left(1\right),d\bar{m}\left(1\right)\rangle$, the expression of $\delta E_{img}\left(v\right)$ in terms of *A*, *B* and *C* is given by

(52) $$\delta E_{img}\left(v\right)=-\frac{1}{\sigma^{2}}\frac{2A}{BC}\langle {\bar{I}}_{1}-\frac{A}{B}\bar{m}\left(1\right),d\bar{m}\left(1\right)\rangle ,$$

yielding

(53) $$\lambda \left(1\right)=\frac{1}{\sigma^{2}}\frac{2}{C}\left(\frac{A}{B}{\bar{I}}_{1}-{\left(\frac{A}{B}\right)}^{2}\bar{m}\left(1\right)\right).$$

By the differentiation of λ(1), the expression of δλ(1) is given by

(54) $$\delta \lambda \left(1\right)=\frac{1}{\sigma^{2}}\frac{2}{C}\left(\Theta {\bar{I}}_{1}-2\frac{A}{B}\Theta \bar{m}\left(1\right)-{\left(\frac{A}{B}\right)}^{2}\right)d\bar{m}\left(1\right),$$

where Θ represents the differential of $\frac{A}{B}$, $\Theta =\frac{{\bar{I}}_{1}-2\frac{A}{B}\bar{m}\left(1\right)}{B}$.

### 3.3. Local Normalized Cross-Correlation (lNCC)

The lNCC image similarity metric departs from the NCC metric by computing the scalar products and the average of images in a neighborhood of size ν

(55) $$E_{img}\left(v\right)=\frac{1}{\sigma^{2}}\int 1-\left(\frac{{\langle \bar{m}\left(1\right),{\bar{I}}_{1}\rangle }_{\nu }^{2}}{∥\bar{m}{\left(1\right)∥}_{\nu }^{2}\cdot {∥{\bar{I}}_{1}∥}_{\nu }^{2}}\right)\left(x\right)d\Omega .$$

The expressions of λ(1) and δλ(1) are the restrictions of Equations (53) and (54) to the ν-neighborhood.

### 3.4. Normalized Gradient Fields (NGFs)

The NGFs image similarity metric is defined as

(56) $$E_{img}\left(v\right)=\frac{1}{\sigma^{2}}\int_{\Omega }1-{\left(\frac{{\langle \nabla m\left(1\right),\nabla I_{1}\rangle }_{\nu \mu }}{{∥\nabla m\left(1\right)∥}_{\nu }{∥\nabla I_{1}∥}_{\mu }}\right)}^{2}\left(x\right)d\Omega ,$$

where ${\langle \cdot ,\cdot \rangle }_{\epsilon }=\langle \cdot ,\cdot \rangle +\epsilon^{2}$ and ${∥\cdot ∥}_{\epsilon }=\sqrt{\langle \cdot ,\cdot \rangle +\epsilon^{2}}$. The image similarity metric can be written in the form of

(57) $$E_{img}\left(v\right)=\frac{1}{\sigma^{2}}\int_{\Omega }1-r{\left(x\right)}^{2}d\Omega ,$$

where *r* is the quotient of the ϵ-scalar product and the ϵ-norms. Let us define $A\left(x\right)={\langle \nabla m\left(1\right)\left(x\right),\nabla I_{1}\left(x\right)\rangle }_{\nu \mu }$, $B\left(x\right)={∥\nabla m\left(1\right)\left(x\right)∥}_{\nu }$, and $C\left(x\right)=∥\nabla I_{1}{\left(x\right)∥}_{\mu }$. We use the same variable naming convention as in the NCC case for highlighting the analogies between both metrics. In this case, the residual is given by $r=\frac{A}{B\cdot C}$.

The expression of $\delta E_{img}\left(v\right)$ is given in terms of *r* by

$$\delta E_{img}\left(v\right)=-\langle 2rdr,dm\left(1\right)\rangle .$$

Using the expression of the differential dr,

$$dA=\langle \nabla I_{1},\nabla dm\left(1\right)\rangle ,$$

and

dB=(1/B)〈∇m(1),∇dm(1)〉,

the expression of $\delta E_{img}\left(v\right)$ in terms of *A*, *B* and *C* is given by

(58) $$\delta E_{img}\left(v\right)=-\frac{1}{\sigma^{2}}\frac{2A}{{\left(BC\right)}^{2}}\langle \nabla I_{1}-\frac{A}{B^{2}}\nabla m\left(1\right),\nabla dm\left(1\right)\rangle ,$$

which can be written in the form of Equation (40) using the identity 〈u,∇v〉=〈−∇·u,v〉. Thus,

(59) $$\begin{array}{c} \lambda \left(1\right)=-\frac{2}{\sigma^{2}}\nabla \cdot \left(\frac{A}{{\left(BC\right)}^{2}}\nabla I_{1}-{\left(\frac{A}{B^{2}C}\right)}^{2}\nabla m\left(1\right)\right). \end{array}$$

Since $\frac{\partial^{2}\Psi }{\partial r^{2}}=2$ for the NGF metric

(60) $$\delta \lambda \left(1\right)=\langle \langle \frac{\partial r}{\partial m(1)},dm\left(1\right)\rangle ,2\frac{\partial r}{\partial m(1)}\rangle .$$

The details of the numerical implementation are given in Appendix B.

### 3.5. Mutual Information (MI)

In PDE-LDDMM, the MI image similarity metric is defined as

(61) $$E_{img}\left(v\right)=-\frac{1}{\sigma^{2}}M\left(m\left(1\right),I_{1}\right)=-\frac{1}{\sigma^{2}}\left(H\left(m\left(1\right)\right)+H\left(I_{1}\right)-H\left(m\left(1\right),I_{1}\right)\right)$$

where H is the entropy function. The entropy for a generic image *I*, and a generic pair of images (I,J), is defined, respectively, as

(62) $$H\left(I\right)=-\int p_{I}\left(r\right)\log p_{I}\left(r\right)dr$$

and

(63) $$H\left(I,J\right)=-\;\int \int \;p_{I,J}\left(r,s\right)\log p_{I,J}\left(r,s\right)drds,$$

where *p* represents the estimated marginal and joint intensity distributions of the images, $p_{I}\left(r\right)=P\left(I=r\right)$ and $p_{I,J}\left(r,s\right)=P\left(I=r\cap J=s\right)$. In the following, we discretize the image variables and use the discrete expressions of the integrals.

The expression of $\delta E_{img}\left(v\right)$ is given by [61]

(64) $$\delta E_{img}\left(v\right)=-\frac{1}{\sigma^{2}}\sum_{r,s}\delta p_{m\left(1\right),I_{1}}\left(r,s\right)\left(1+\log\frac{p_{m\left(1\right),I_{1}}\left(r,s\right)}{p_{m(1)}\left(r\right)}\right).$$

In order to compute the differential $\delta p_{m\left(1\right),I_{1}}\left(r,s\right)$, we use the expression of *p* in analytical form

(65) $$p_{m\left(1\right),I_{1}}\left(r,s\right)=\frac{1}{N_{x}}\sum_{x}\xi \left(r-\bar{m}\left(1\right)\left(x\right)\right)\xi \left(s-{\bar{I}}_{1}\left(x\right)\right),$$

yielding

(66) $$\delta p_{m\left(1\right),I_{1}}\left(r,s\right)=\frac{1}{N_{x}}\sum_{x}\delta \xi \left(r-\bar{m}\left(1\right)\left(x\right)\right)\xi \left(s-{\bar{I}}_{1}\left(x\right)\right).$$

We use b-spline functions for the expression of ξ as proposed in [32]. The differential of ξ, δξ, can be computed using the chain rule as

(67) $$\delta \xi \left(r-\bar{m}\left(1\right)\left(x\right)\right)=-\langle \frac{\partial \xi }{\partial r},d\bar{m}\left(1\right)\rangle .$$

Gathering the above expressions yields the expression of the initial adjoint variable

(68) $$\begin{array}{c} \lambda \left(1\right)=-\frac{1}{\sigma^{2}}\sum_{r,s}\frac{1}{N_{x}}\sum_{x}\frac{\partial \xi }{\partial r}\xi \left(s-{\bar{I}}_{1}\left(x\right)\right)\;\;\;\;\;\;\;\;\;\;\;\;\;\;\;\\ \;\;\;\;\;\;\;\;\;\;\;\;\;\;\;\left(1+\log\frac{p_{m\left(1\right),I_{1}}\left(r,s\right)}{p_{m(1)}\left(r\right)}\right). \end{array}$$

In the computation of δλ(1), a huge dense-matrix product requesting more than 5000 GBs of RAM memory arises. Therefore, we restrict this work to the gradient-descent version of PDE-LDDMM for MI. The derivation of δλ(1) needed in Gauss–Newton–Krylov optimization is left outside the scope of the present work.

## 4. Experimental Setup

### 4.1. Datasets

We used five different databases in our evaluation:

**NIREP16**, was proposed in [41] for the evaluation of non-rigid registration. NIREP16 consists of 16 T1 Magnetic Resonance Imaging (MRI) images. NIREP16 images were acquired at the Human Neuroanatomy and Neuroimaging Laboratory, University of Iowa. They were selected for the NIREP project from a database of 240 normal volunteers. Datasets correspond to 8 males and 8 females with a mean age of 32.5±8.4 and 29.8±5.8 years, respectively. The images are skull stripped and aligned according to the anterior and posterior commissures. Image dimension is 256×300×256 with a voxel size of 0.7×0.7×0.7 mm. Images are distributed with the segmentation of 32 gray matter regions at frontal, parietal, temporal, and occipital lobes. The most remarkable feature of this dataset is its excellent image quality. The geometry of the segmentations provides a specially challenging framework for deformable registration evaluation. In our previous works, a subsampled version of this dataset has been extensively used for the evaluation of different LDDMM methods. The mages of this dataset have been subsampled by reducing image dimension to 180×210×180 with a voxel size of 1.0×1.0×1.0 mm. Subsampling is needed to be able to run interesting but memory-demanding benchmark methods and to maintain the continuity of the evaluation results shown in previous works.

**Klein datasets** were proposed in [42] in the first extensive evaluation study of non-rigid registration methods. The datasets contain the T1 MRI images and segmentations from the LPBA40, IBSR18, CUMC12, and MGH10 databases. The four databases provide images with different levels of quality, providing varying difficulties for deformable registration [62]. Image dimension is 182×218×182 with a voxel size of 1.0×1.0×1.0 mm.

*LPBA40* contains 40 skull-stripped brain images without the cerebellum and the brain stem. LPBA40 provides the segmentation of 50 gray matter structures together with left and right caudate, putamen, and hippocampus. LPBA40 protocols can be found at https://loni.usc.edu/research/atlases (accessed on 1 January 2022).

*IBSR18* contains 18 brain images with the segmentation of 96 cerebral structures. This dataset provides the segmentation of brain structures of interest for the evaluation of image registration methods. The image quality is low. For example, most of the images show motion artifacts. The variability of the ventricle sizes is high.

*CUMC12* contains 12 full brain images with the segmentation of 130 cerebral structures. Overall, the image quality is acceptable, although some of the images are noisy. The variability of the ventricle sizes is high.

*MGH10* contains 10 full brain images with the segmentation of 106 cerebral structures. Overall, the image quality is acceptable, although some of the images are noisy. Ventricle sizes are usually all big.

In addition, we studied the performance of our methods in a multi-modal experiment, where the images were obtained from:

**Oasis.** The open-access series of imaging studies (https://www.oasis-brains.org/, accessed on 1 January 2022) is a project aimed at making neuroimaging data sets of the brain freely available to the scientific community. OASIS-3 compiles images from more than 1000 participants ranging from cognitively normal to various stages of cognitive decline. For each participant, the study includes different MRI sessions including T1, T2, FLAIR, and others. Our multimodal experiment selected a T2 image from an Alzheimer’s disease participant as the source, and a T1 image from a cognitive normal participant as the target image.

### 4.2. Image Registration Pipeline

The evaluation in NIREP16 was performed consistently with our previous works on PDE-LDDMM diffeomorphic registration. The registrations were carried out from the first subject to every other subject in the database, yielding a total of 15 registrations per variant, optimization method, and image similarity metric. The subsampled NIREP16 database was obtained from the resampling of the original images into volumes of size 180×210×180 with a voxel size of 1.0×1.0×1.0 mm after the alignment to a common coordinate system using affine transformations. The images were scaled between 0 and 1 for SSD and NCC metrics, and between 0 and 255 for lNCC, NGFs, and MI. The affine alignment and subsampling were performed using the Insight Toolkit (ITK).

The LPBA40, IBSR18, CUMC12, and MGH10 images were preprocessed similarly to [42]. The input images were selected from the Synapse repository (https://www.synapse.org/#%21Synapse:syn3217707, accessed on 1 January 2022 ) in the folder hosting FLIRT affine registered images. In the first place, histogram matching was applied to all the images. The images were then scaled between 0 and 1 for SSD and NCC metrics, and between 0 and 255 for lNCC, NGFs, and MI. To perform these preprocessing steps we used the algorithms available in ITK.

Oasis images were finely aligned to the MNI152 atlas with NiftyReg and then skull stripped using the robust brain extraction software RobEx (https://www.nitrc.org/projects/robex, accessed on 1 January 2022).

### 4.3. Numerical Details, Parameter Configuration, and Implementation Details

The experiments were run on a cluster of two machines equipped with four NVidia GeForce GTX 1080 ti with 11 GBS of video memory and an Intel Core i7 with 64 GBS of DDR3 RAM, and two NVidia Titan RTX with 24 GBS of video memory and an Intel Core i7 with 64 GBS of DDR3 RAM, respectively. The codes were developed in the GPU with Matlab 2017a and Cuda 8.0.

Regularization parameters were selected from a search of the optimal parameters in the registration experiments performed in our previous work [22]. We selected the parameters $\sigma^{2}=1.0$, α=0.0025, and s=2 and a unit-domain discretization of the image domain Ω [56]. We also tested the parameters $\sigma^{2}=0.03$, α=3.0, and s=3 and a spatial-domain discretization of Ω, selected as optimal in [58]. For gradient-descent optimization, we obtained excellent evaluation results; however, the obtained maximum Jacobians were much higher than recommended. On the other hand, Gauss–Newton–Krylov showed convergence problems during PCG, with negative curvature values found at early inner iterations. This suggests that the specific selection of parameters in [58] might achieve fairly high structural overlaps with the cost of very aggressive underlying deformations which are glimpsed in the malfunctioning of Gauss–Newton.

The BL experiments were performed with band sizes of 32×32×32 for BL Variants I and II. This selection was found as optimal for each method in our previous work [20,22,33].

Gradient-descent was implemented with an efficient method for the update of the step size based on offline backtracking line-search combined with a check on Armijo’s condition. We used the stopping conditions in [13,32]. Otherwise, the optimization was stopped after 50 iterations.

Gauss–Newton–Krylov was also implemented with an offline backtracking line-search combined with a check on Armijo’s condition. The number of PCG iterations was set to five. The PCG tolerance was selected from

$$\tau =\min\left(0.5,\sqrt{\frac{∥\nabla_{v}E\left(v^{n}\right){∥}_{2}}{∥\nabla_{v}E\left(v^{0}\right){∥}_{2}}}\right).$$

We used the stopping conditions in [13,32]. Otherwise, the optimization was stopped after 10 iterations. These parameters were selected as optimal in our previous work since the methods achieved state-of-the-art accuracy in a reasonable amount of time [22].

PDE-LDDMM was embedded into a multi-resolution scheme. The images were subsampled, and the velocity fields were resampled similarly to [63,64]. The PDE-LDDMM registration methods were executed on each resolution level. For the multi-resolution experiments, the pyramid was built with three levels with the same number of outer and inner iterations, as for the single-resolution.

To integrate the PDEs, we used the semi-Lagrangian Runge–Kutta schemes proposed in [33] for the SSD versions of Variants I and II. The solutions were computed at the Chebyshev–Gauss–Lobatto discretization of the temporal domain [0,1]. The number of time steps was selected as five. Since Matlab lacks a 3D GPU cubic interpolator, we implemented in a Cuda MEX file the GPU cubic interpolator with prefiltering proposed in [65].

The computation of differentials was approached using Fourier spectral methods as a machine-precision accurate alternative to commonly used finite difference approximations [66]. Spectral methods allow solving of ODEs and PDEs for high accuracy in simple domains for problems involving smooth data. To this end, the images were smoothed with a Gaussian filter as a preprocessing step. However, for the Gauss–Newton–Krylov version of NGFs, we used the matrix version of the differential operators, and then the computation of differentials must be approached with finite difference approximations. To be consistent with the input data, the images were also smoothed as a preprocessing step.

For lNCC, the size of the neighborhood was selected as four. For NGFs, the value of $\epsilon^{2}$ for the ϵ-norms was equal to 1000. For MI, the number of histogram bins was selected equal to 16. The computation of the adjoint variable for MI required the use of sparse matrices and was implemented in the CPU since Matlab does not yet have GPU support for these data structures.

### 4.4. Benchmarks

For benchmarking, we run single- and multi-resolution versions of ANTS registration with SSD, lNCC and MI image similarities [40]. We also extended Stationary LDDMM (St-LDDMM), proposed in [67] as an efficient stationary variant of Beg et al.’s LDDMM [56], with NCC, lNCC, NGFs, and MI metrics. The details of the method extension can be found in Appendix A. In addition, we studied the accuracy obtained with QuickSilver, a supervised deep-learning based method with SSD in the loss function [46], and VoxelMorph, an unsupervised deep learning-based model with SSD and NCC metrics in the loss function [50].

St-LDDMM was run with the same parameters than PDE-LDDMM in the common steps of the algorithms. ANTS was run with the following parameters for the single-resolution experiments

$synconvergence = “[50,$1\times 10^{-6}$,10]”,
$synshrinkfactors = “1”,
and $synsmoothingsigmas = “3vox”.  

For the multi-resolution experiments the parameters were set to

$synconvergence = “[50×50×50,$1\times 10^{-6}$,10]”,
$synshrinkfactors = “4×2×1”,
and $synsmoothingsigmas = “3×2×1vox”.

The selection of the number of iterations was in agreement with the number of iterations used in gradient-descent and the number of outer × inner iterations used in Gauss–Newton–Krylov optimization for PDE-LDDMM.

In the multi-modal experiment, we compare our proposed methods with NiftyReg, a software for efficient registration developed at the Centre for Medical Image Computing at University College London, UK (https://sourceforge.net/projects/niftyreg/, accessed on 1 January 2022). NiftyReg is usually selected as a benchmark for non-rigid multimodal image registration. We also include in the comparison ANTS and SynthMorph, a VoxelMorph adaptation for building deep-learning models capable of dealing with multimodality [53].

### 4.5. Metrics and Statistical Analysis for Registration Evaluation

The evaluation in NIREP16 and Klein datases is based on the accuracy of the registration results for template-based segmentation. The Dice Similarity Coefficient (DSC) is selected as the evaluation metric. Given two segmentations *S* and *T*, the DSC is defined as

(69) $$DSC\left(S,T\right)=\frac{2Vol(S\cap T)}{Vol(S)+Vol(T)}.$$

This metric provides the value of one if *S* and *T* exactly overlap and gradually decreases towards zero depending on the overlap of the two volumes. The statistical distribution of the DSC results across the segmented structures are shown in the shape of box and whisker plots following the evaluation methods in [42]. The DSC distribution is taken over the DSC values over the different segmentation labels. This way of computing the DSC distribution reflects the recommendation given in [68] that the evaluation of non-rigid registration with the segmentation of sufficiently locally labeled regions of interest is strongly recommended for obtaining reliable measurements of the performance of the registration.

The evaluation in NIREP16 was completed with two different statistical analysis on the DSC values. In the first place, the analysis of variance (ANOVA) was conducted in order to assess whether the means of the DSC distributions are different for the image similarity metrics when observations are grouped by type of method (baseline vs. PDE-LDDMM) or variants (I vs. II). Baseline methods include ANTS, Stationary LDDMM, VoxelMorph, and QuickSilver. In the second place, pairwise right-tailed Wilcoxon rank-sum tests were conducted for the assessment of the statistical significance of the difference of medians for the distribution of the DSC values. The alternative hypothesis is that the median of the first distribution is higher than the median of the second one.

Finally, we include for NIREP16 the quantitative assessment provided by the mean and standard deviation of the relative image similarity error after registration,

$$MSE_{rel}=\frac{∥m\left(1\right)-I_{1}{∥}_{L^{2}}^{2}}{∥I_{0}-I_{1}{∥}_{L^{2}}^{2}},$$

the relative gradient magnitude,

$${∥g∥}_{\infty ,rel}=\frac{∥\nabla_{v}E\left(v^{n}\right){∥}_{\infty }}{∥\nabla_{v}E\left(v^{0}\right){∥}_{\infty }},$$

and the extrema of the Jacobian determinant.

## 5. Results

In this section, we show the experiments conducted to evaluate the performance of the two PDE-LDDMM variants for the different image similarity metrics. First, we provide an extensive evaluation of our proposed methods in the NIREP16 database, where we have extensively evaluated previous LDDMM and PDE-LDDMM registration methods [20,22,33,67]. Next, we evaluate our proposed methods in Klein et al. databases. Finally, we compare the behavior of the different metrics in a challenging multimodal experiment.

### 5.1. Results on NIREP16

#### 5.1.1. Evaluation

**Figure 1:**
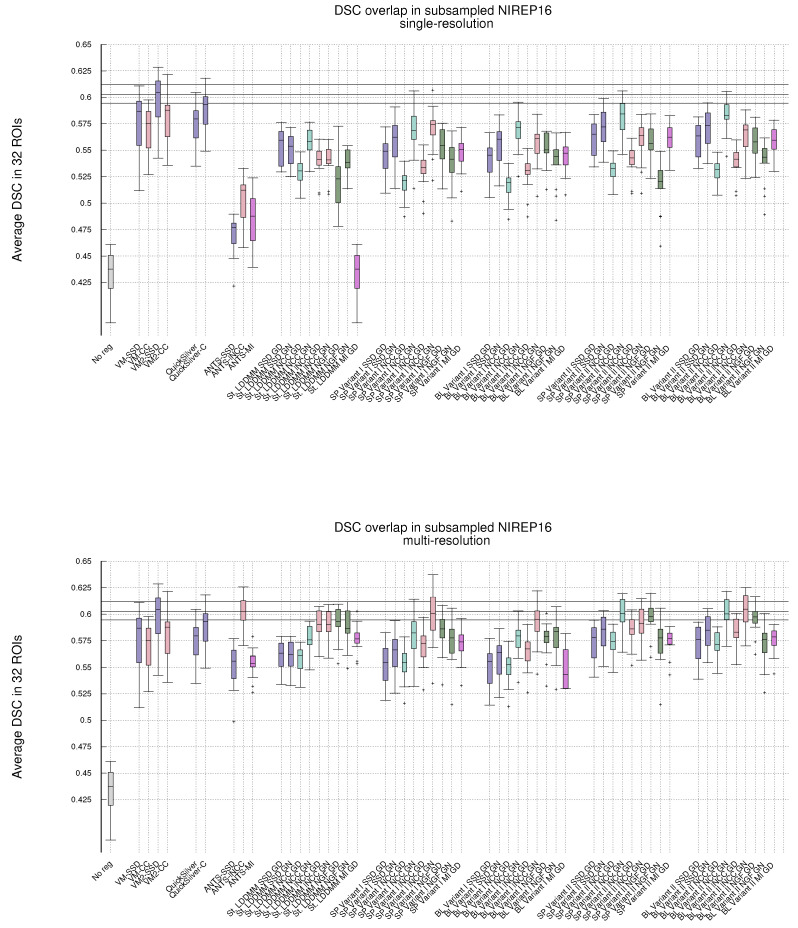
NIREP16. Volume overlap obtained by the registration methods measured in terms of the DSC between the warped and the corresponding manual target segmentations. Box and whisker plots show the distribution of the DSC values averaged over the 32 NIREP manual segmentations. The whiskers indicate the minimum and maximum of the DSC values. The upper plot shows the evaluation results in the single-resolution experiments. The lower plot shows the results in the multi-resolution experiments. The horizontal lines in the plot indicate the first, second, and third quartiles of one of the best-performing baseline methods (multiresolution ANTS-lNCC), facilitating the comparison.

**Figure 2:**
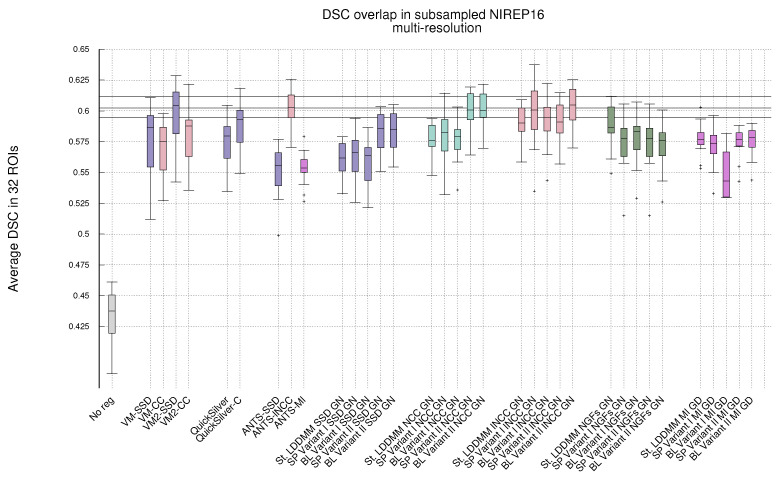
NIREP16. Same legend to Figure 1. Results obtained with PDE-LDDMM and Gauss–Newton–Krylov optimization. Boxplots grouped by variant. St-LDDMM is grouped with PDE-LDDMM results for facilitating comparison.

Figure 1 shows, in the shape of box and whisker plots, the statistical distribution of the DSC values that were obtained after the registration across the 32 segmented structures. In addition, Figure 2 gathers the results obtained with Gauss–Newton–Krylov optimization grouped by variant for a better assessment of the best-performing combination of variant and metrics. Our first observation is that the DSC coefficients for the multi-resolution experiments outperform the single-resolution experiments. The improvement is substantial for lNCC, NGFs, and MI metrics.

**Single-resolution**. Regarding the single-resolution experiments, it is striking the low performance of ANTS for all metrics. Both St-LDDMM and PDE-LDDMM perform more reasonably than ANTS. In general, PDE-LDDMM methods tend to outperform St-LDDMM.

For PDE-LDDMM and SSD, the differences between Gauss–Newton and gradient-descent are small. Gauss–Newton optimization significantly outperforms gradient-descent for NCC and lNCC. On the contrary, for NGFs, gradient-descent optimization outperforms Gauss–Newton in all cases. The spatial version of Variant II exhibits an especially lower performance. For St-LDDMM, the trends observed in PDE-LDDMM are also observed for SSD and NCC metrics. However, gradient-descent performs similarly to Gauss–Newton for lNCC and Gauss–Newton outperforms gradient descent for the NGFs metric.

The results obtained with the spatial versions of the PDE-LDDMM variants are similar to the corresponding BL versions. Comparing the accuracy of both variants, Variant II provides in general better performance than Variant I.

For all variants, the overall best performing metric is NCC. For almost all variants, the gradient-descent version of NGFs performs similarly to the Gauss–Newton version of lNCC. For the BL methods, the Gauss–Newton version of NGFs also performs similarly to the gradient-descent version of SSD. Moreover, the gradient-descent version of MI performs similarly to the gradient-descent version of SSD.

**Multi-resolution**. Regarding the multi-resolution experiments, the performance of ANTS reached the level of accuracy of St-LDDMM and the PDE-LDDMM methods. ANTS with the lNCC metric greatly outperformed the other ANTS variants using SSD and MI, ranking among the best-performing methods. In general, PDE-LDDMM methods tend to outperform St-LDDMM, with the exception of the NGFs metric.

As happened with the single-resolution experiments for PDE-LDDMM, the differences between Gauss–Newton optimization and gradient-descent are small for the SSD metric. Gauss–Newton also outperforms gradient-descent for NCC and lNCC. For NGFs, gradient-descent optimization greatly outperforms Gauss–Newton in the case of Variant II. However, for Variant I, the differences between both optimization methods are small, especially for the BL version of the methods. The performance of the NGFs metric is further explored in Appendix B for a better understanding of these observations. For St-LDDMM, the trends observed with the single-resolution experiments are mostly observed.

For Variant I, the spatial version tends to outperform the BL version of the same variant slightly. However, the performance of the BL version of Variant II is similar or even improves the spatial version for almost all metrics. As happened with the single-resolution experiments, Variant II provides better performance than Variant I. The best performing metric for Variant I is lNCC, while for Variant II, the best-performing metric is still NCC, closely followed by lNCC. The resemblance of performance between MI and SSD metrics in the single-resolution experiments remains for the multi-resolution experiments. However, the excellent performance of NGFs metric with gradient-descent optimization is remarkable, ranking close to the best-performing metrics for Variant II.

Comparing ANTS with PDE-LDDMM methods, Variant I with SSD and gradient-descent performs similarly to ANTS-SSD. In the case of MI, PDE-LDDMM methods outperform ANTS-MI. Some PDE-LDDMM methods achieve results competing with ANTS-lNCC for the NCC and lNCC metrics.

**Deep-learning methods**. Because of the increasing relevance of deep-learning methods in the field, we added to our evaluation the performance of VoxelMorph [50] and QuickSilver [46]. VoxelMorph with SSD and QuickSilver with the correction step performed similarly to Variant II of PDE-LDDMM with the SSD metric. Diffeomorphic VoxelMorph with SSD ranked among the best-performing methods, with a box-plot distribution similar to Variant I with lNCC and BL Variant II with NCC and lNCC. Despite all LDDMM methods agreeing in the much better performance of NCC and lNCC metrics over SSD, Diffeomorphic VoxelMorph trained with a loss function based on SSD greatly outperformed the method trained with NCC. Lastly, it is a remarkable fact that, although VoxelMorph is informed during training of the performance through the DSC, our best-performing PDE-LDDMMs were able to achieve competitive results without the use of this information.

**Statistical analysis**. Table 1 shows the results of the analysis of variance (ANOVA) for the effects of method and image similarity metric selection on the distribution of the DSC values obtained in the multi-resolution experiments with Gauss–Newton optimization (with the exception of the methods combined with the MI metric). The methods on the first factor were grouped by type of method (baseline vs. PDE-LDDMM) and variants. The tests only showed no statistical significance for the differences between the spatial versions of Variant I and Variant II. The selection of the $E_{i}mg$ metric resulted in statistical significance for all cases.

**Figure 3:**
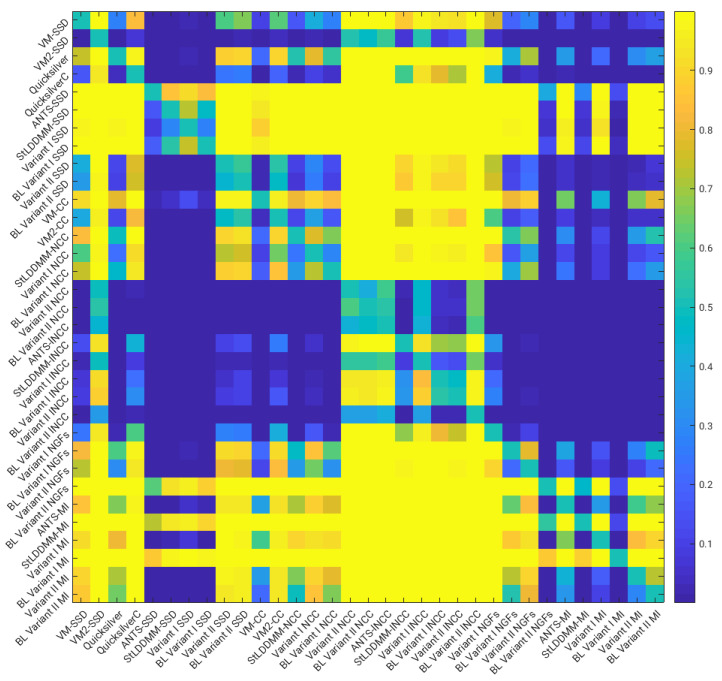
NIREP16. Multi-resolution experiments and Gauss–Newton–Krylov optimization. Results of the pairwise right-tailed Wilcoxon rank-sum tests.

Figure 3 shows the *p*-values of pairwise right-tailed Wilcoxon rank-sum tests for the distribution of the DSC values obtained in the multi-resolution experiments with Gauss–Newton optimization (with the exception of the methods combined with the MI metric). The figure shows statistical significance for the better performance of the NCC and lNCC metrics over SSD and MI. For NGFs, obtaining statistical significance depends on the method. Among the best-performing methods, no statistical significance was found for the difference of medians.

#### 5.1.2. Quantitative Assessment

Table 2 shows, averaged by the number of experiments, the mean and standard deviation of the $MSE_{rel}$, ${∥g∥}_{\infty ,rel}$, and the extrema of the Jacobian determinant obtained with PDE-LDDMM in the NIREP16 dataset. We restrict the results to the methods with Gauss–Newton–Krylov optimization with the exception of the methods with the MI metric. For NGFs, the results with gradient descent and different Gauss–Newton approximations are analyzed in depth in Appendix B. Table 3 shows the average $MSE_{rel}$ values and the extrema of the Jacobian determinant for VoxelMorph and QuickSilver.

For the single-resolution experiments, the best $MSE_{rel}$ values were obtained by the NCC metric, followed by SSD. Although the $MSE_{rel}$ values for the lNCC and MI metrics ranged higher than 20%, their performance in the evaluation reported a similar distribution. For lNCC, NGFs, and MI, the correlation between the lowest $MSE_{rel}$ values and the highest DSC results that are usually seen for SSD in previous works does not hold anymore [22,33].

The spatial methods slightly outperformed the BL methods in terms of the $MSE_{rel}$ values, as expected. Variant II performed better than Variant I. The relative gradient was reduced to average values ranging from 0.01 to 0.18, except for the lNCC and NGFs metrics. This means that the optimization was stopped in acceptable energy values in all these cases. Although the relative gradient obtained with lNCC was higher than recommended, the corresponding DSC distributions indicate that the lNCC methods can reach a local minimum providing good registration results. All the Jacobians remained above zero.

For the multi-resolution experiments, the results regarding the $MSE_{rel}$ values and the Jacobians were consistent with the single-resolution experiments. However, the high values of the relative gradient indicate a stagnation of the convergence in the finer resolution level that may be due to the method already starting close to the convergence point at the beginning of this resolution level.

Both VoxelMorph and QuickSilver usually obtained $MSE_{rel}$ values greater than PDE-LDDMM with the corresponding image similarity metric. It is striking the magnitude of the Jacobian extrema obtained by VoxelMorph and its diffeomorphic version, indicating that the accuracy of the registration results shown in Figure 2 are obtained through large folds in a considerable number of locations.

**Figure 4:**
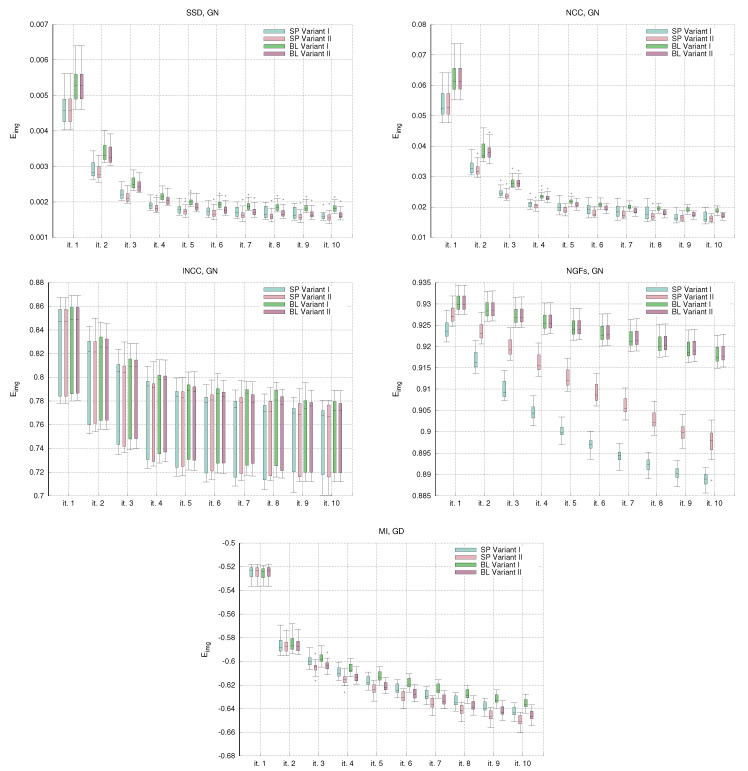
NIREP16. Single-resolution experiments. Box and whisker plots of the $E_{img}$ convergence values in the single resolution experiments. For gradient-descent, the energy values are shown every five iterations.

Figure 4 shows the evolution of the convergence curves for the image similarity metrics $E_{img}$ in the single-resolution experiments. For all the metrics, the trend of the $E_{img}$ values is decreasing. The most unexpected behavior is for the curves of the lNCC metrics, where the standard deviation remains stable and large in comparison with the energy reduction. The curves of the NGFs metrics show the stagnation of the energy values for the BL variants. This is the cause of the low DSC distributions already shown in Figure 1.

Spatial methods show slightly better $E_{img}$ values than BL methods. Comparing the variants, Variant II provides slightly lower $E_{img}$ values than Variant I. These results are consistent with the evaluation and the quantitative assessment shown in Figure 1 and Table 2.

#### 5.1.3. Qualitative Assessment

**Figure 5:**
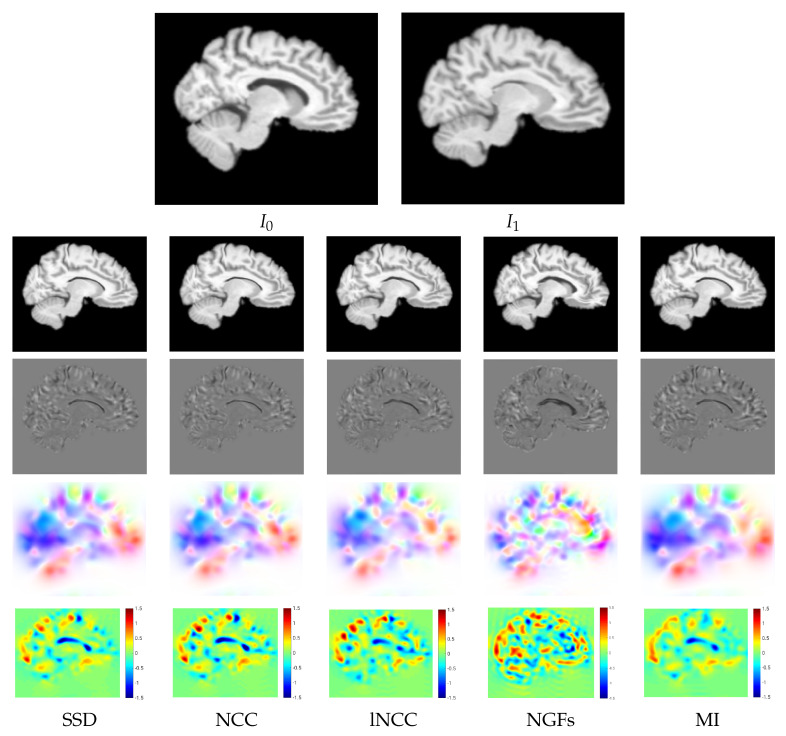
NIREP16. BL Variant II of PDE-LDDMM. Multi-resolution experiments. Sagittal view of the warped sources, the intensity differences after registration, the velocity fields, and the logarithm of the Jacobian determinants after registration for the different image similarity metrics. The results for SSD, NCC, lNCC, and NGFs are obtained with Gauss–Newton optimization, while for MI gradient-descent is used.

**Figure 6:**
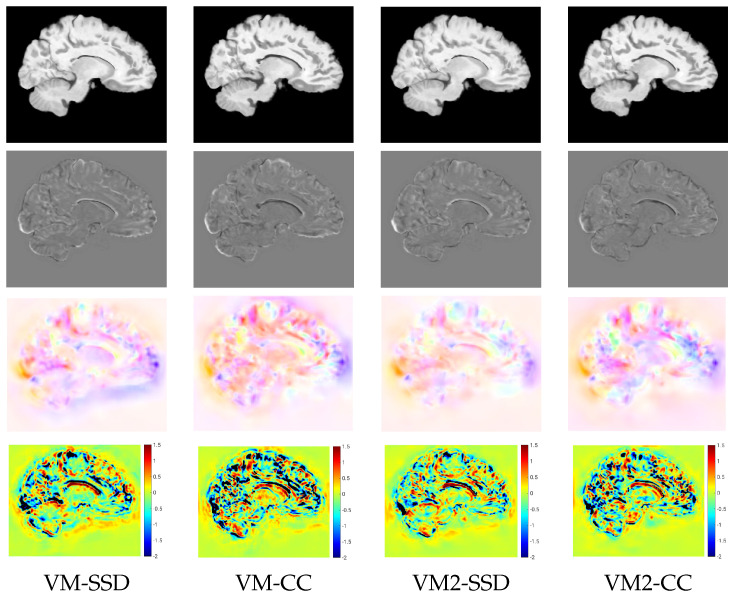
NIREP16. VoxelMorph experiments. Sagittal view of the warped sources, the intensity differences after registration, the displacement fields, and the logarithm of the Jacobian determinants after registration for the different image similarity metrics. For the negative Jacobian values, the logarithm is replaced by −2 and displayed in black.

For a qualitative assessment of the proposed registration methods, we show the registration results obtained by the different metrics for the BL version of Variant II in the multi-resolution experiments. Figure 5 shows the warped images, the difference between the warped and the target images after registration, the velocity fields, and the logarithm of the Jacobian determinant. The resemblance of the differences between the warped and the target images was high for all the metrics except for NGFs. Focusing on the registration results at the ventricle, SDD and NCC were able to achieve the best compression of the structure, while NGFs obtained the worst registration results at this location. Figure 6 shows the warped images, the difference between the warped and the target images, the displacement fields, and the logarithm of the Jacobian determinant for VoxelMorph. The resemblance of the differences between the warped and the target images was higher for SSD than NCC. The displacement fields were visually less smooth than the velocity fields obtained with PDE-LDDMM. The Jacobian determinant had negative regions all over the image. In particular, the registration results at the ventricle were achieved through large expansions and foldings in its upper boundary.

#### 5.1.4. Computational Complexity

Table 4 shows the average and standard deviation of the total computation time and the VRAM peak memory reached through the computations in the NIREP16 database for the single-resolution experiments. The BL methods achieved a substantial time and memory reduction over the spatial methods, as already demonstrated in [22,33,39]. From the Gauss–Newton methods, the methods with SSD and NCC image similarity metrics were the most efficient ones, as expected. On the other side, the methods with MI were the most time-consuming ones. Regarding memory usage, the methods using SSD and NCC were more efficient than lNCC. The memory efficiency shown by NGFs and MI metrics was due to the combination with gradient-descent and the need to perform operations involving sparse matrices on the CPU.

### 5.2. LPBA40, IBSR18, CUMC12, and MGH10 Evaluation Results

**Figure 7:**
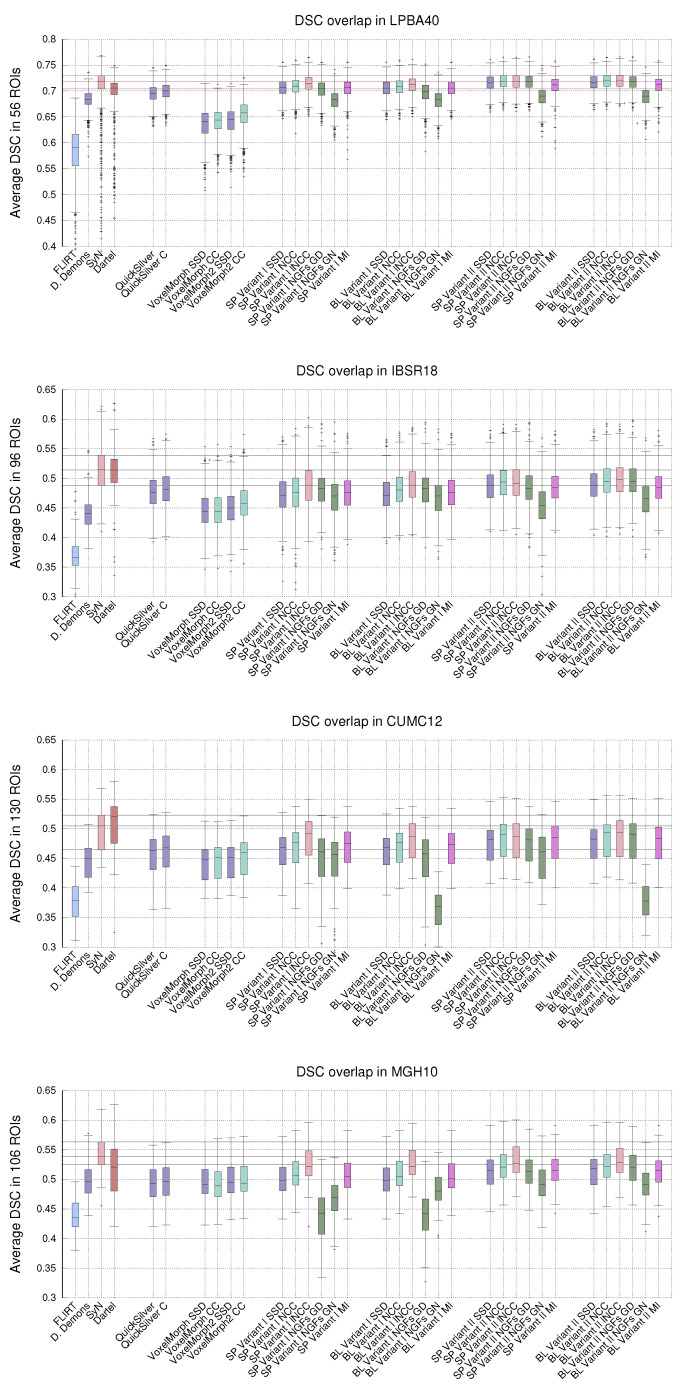
LPBA40, IBSR18, CUMC12, and MGH10. Distribution of the DSC values averaged over the manual segmentations in the registration experiments. The whiskers indicate the minimum and maximum of the DSC values. The horizontal lines indicate the first, second, and third quartiles of the SyN benchmark method, facilitating the comparison.

Figure 7 shows the statistical distribution of the DSC values obtained with PDE-LDDMM for Klein databases [42]. As a benchmark, we include the results reported in [42] for affine registration (FLIRT) and three diffeomorphic registration methods: Diffeomorphic Demons, SyN, and Dartel. We also include the results of QuickSilver and VoxelMorph.

For LPBA40, Variant I with lNCC and Variant II with metrics from SSD to NGFs reached a performance similar to SyN with many outliers significantly reduced. For each metric, Variant II outperformed the corresponding Variant I. The worst performing results were consistently achieved by NGFs and Gauss–Newton–Krylov optimization. QuickSilver performed slightly better under SDD and NCC versions of Variant I. VoxelMorph was the worst-performing method for all metrics.

For IBSR18, SP and BL Variant I with lNCC, SP Variant II with NCC and BL Variant II with NCC and lNCC metrics were the best performing PDE-LDDMM methods. Their performance was slightly over the one exhibited by QuickSilver and greatly over the one obtained with VoxelMorph. However, in all cases, these methods underperformed SyN and Dartel.

For CUMC12, the best performing PDE-LDDMM methods were SP and BL Variant I with lNCC and Variant II with NCC and lNCC. As happened with IBSR18, these methods slightly outperformed QuickSilver while greatly outperformed VoxelMorph. It is remarkable the low performance of BL variants with NGF and Gauss–Newton–Krylov optimization. All the methods underperformed SyN and Dartel methods.

Finally, for MGH10, the best performance was achieved by variants I and II with lNCC similarity metric. It is remarkable the low performance of Variant I with NGF with gradient descent underperforming Gauss–Newton–Krylov optimizers. In this case, the methods underperformed SyN, while the best-performing methods showed a DSC distribution similar to Dartel. QuickSilver and VoxelMorph achieved performance similar to the SSD version of Variant I.

These results corroborate the better performance of Variant II over Variant I obtained in the evaluation with NIREP16 for the majority of metrics. The lNCC metric is positioned as the best-performing one for the majority of methods and databases. The NGFs metric has shown better performance for gradient descent optimization in the great majority of experiments. The best PDE-LDDMM combination of variants and metrics overpassed deep-learning based methods in all the datasets.

Regarding the consistent outperformance of SyN and Dartel over all the considered methods, we found out that SyN used a probabilistic image similarity metric while Dartel used tissue probability maps as inputs. The images in IBSR18, CUMC12, and MGH10 have low contrast, and, therefore, the algorithmic choices performed by SyN and Dartel overpass the use of challenging inputs. We have also seen that performing histogram equalization for contrast enhancement as in QuickSilver original paper [46] improved the evaluation results reaching SyN and Dartel performance. However, this preprocessing reduces the influence of the used metrics in the obtained DSCs and provides less informative results.

## 6. Multimodal Experiment

**Figure 8:**
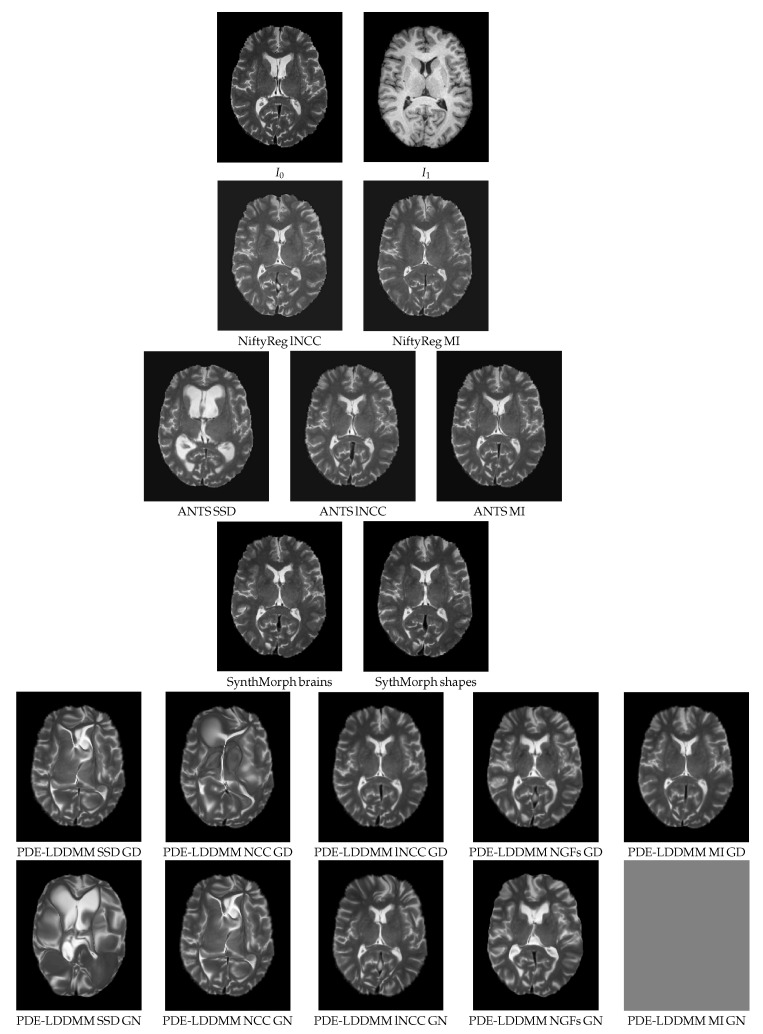
Oasis. BL PDE-LDDMM Variant II. Warped sources obtained for the methods considered in the multimodal simulated experiment.

Figure 8 shows an axial view of the registration results obtained by NiftyReg, ANTS, SynthMorph, and BL PDE-LDDMM Variant II in the Oasis multimodal experiment. The worst-performing metrics are SSD (as expected) and NCC. All methods with lNCC, NGFs, and MI metrics provide acceptable registration results with subtle differences between the warps located at the ventricle front horns and the atrium and the distribution of gyri and sulci in the cerebral cortex. The most visually accurate methods are NiftyReg, ANTS-MI, SynthMorph, and PDE-LDDMM from lNCC and MI. It should be noticed that the registration results of NiftyReg are obtained at the cost of folding the transformations. SynthMorph provides excellent registration results but the used similarity metric is DSC over the segmented images, overpassing the direct use of images from different modalities.

## 7. Discussion and Conclusions

In this work, we have presented a unifying framework for introducing different image similarity metrics in the two best-performing variants of PDE-LDDMM with Gauss–Newton–Krylov optimization [22,33]. From the Lagrangian variational problem, we have identified that the change in the image similarity metric involves changing the initial adjoint and the initial incremental adjoint variables. We derived the equations of these variables for NCC, its local version (lNCC), NGFs, and MI. PDE-LDDMM with Gauss–Newton–Krylov optimization being successfully extended from SSD to NCC, and lNCC image similarity metrics. For NGFs, the method was not able to overpass gradient-descent optimization. With MI, the computation of the Hessian-matrix product required the product of dense matrices that requested more than 5000 GBs of memory, thus becoming far from feasible. Therefore, we obtained varying degrees of success in our initial objective.

The evaluation performed in NIREP16 database has shown the superiority of Variant II with respect to Variant I, as happened in [22,33]. In addition, the results reported for the BL version of Variant II were statistically indistinguishable from the SP (spatial) version. For any image similarity metric, BL Variant II overpassed the baseline established by ANTS. For BL Variant II, NCC and its local version were the best-performing metrics, closely followed by the gradient-descent version of NGFs. The superiority of these metrics was statistically significant. The outperformance of lNCC was quantified for the first time for ANTS diffeomorphic registration with gradient-descent and LPBA40 in [36]. Our best-performing variants overpassed QuickSilver, a supervised deep-learning method for diffeomorphic registration. In addition, they provided competitive results when compared with VoxelMorph with the added value of PDE-LDDMM being agnostic to the evaluation metric and providing purely diffeomorphic solutions.

The $MSE_{rel}$ values were in agreement with the DSC distributions obtained with NCC and SSD. However, for lNCC, NGFs and MI, the correlation between the $MSE_{rel}$ values and the DSC seen usually for SSD in previous works does not hold anymore.

The experiments with Klein databases corroborated the superiority of Variant II over Variant I for almost all the metrics. The evaluation in LPBA40 has shown how PDE-LDDMM based on the deformation state equation performs similarly to SyN for the majority of metrics with a reduced number of outliers. The evaluation in IBSR18, CUMC12, and MGH10 datasets has consistently shown lNCC as the best-performing metric for PDE-LDDMM. It is striking that the optimum DSC values greatly vary depending on the dataset used for evaluation. For example, SyN obtains an average DSC value greather than 0.7 for LPBA40 while the average DSC value is close to 0.5 for IBSR18, CUMC12, and MGH10 data. We believe that the disparity of the obtained DSC values depends on the geometry of the anatomies involved in the dataset which may downgrade the overall accuracy.

Although not being able to report functional Gauss–Newton–Krylov PDE-LDDMM methods for NGFs and MI has been disappointing, it encourages us to embed PDE-LDDMM into different optimization methods competing with gradient-descent as Gauss–Newton–Krylov does for the SSD, NCC, and lNCC metrics. In future work, we will address the problem with limited-memory BFGS or, in the framework of Krylov subspace methods, with the generalized minimal residual method (GMRES).

Our method has shown visually acceptable registration results on a challenging multi-modal intra-subject experiment. The results were competitive with SynthMorph, a deep-learning method that uses a loss function based on DSC from image segmentations. The experiment pointed out the differences between the combination of different optimization methods and metrics. In future work, we will explore in depth the influence of metric and optimization selection in the accuracy of multi-modal registration.

Despite the methodological improvements that have been subsequently proposed in PDE-LDDMM for efficiency (Gauss–Newton–Krylov optimization, band-limited parameterization, and Semi-Lagrangian Runge–Kutta integration), our PDE-LDDMM methods are able to compute a diffeomorphism in a volume of size 180×210×180 in one to five minutes, depending on the variant and the metric. This may be considered a non-acceptable amount of time in comparison with modern deep-learning approaches where the inference takes about one second. However, the time and resources needed for training are not usually considered in the comparison while they should be at least apportioned. In addition, deep-learning methods are not memory efficient while our proposed methods run in a commodity graphics card with a VRAM of less than 4 GBs.

BL Variant II with SSD, NCC, and lNCC has been recently included in the diffeomorphic normalization step into the pipeline of Spasov et al. [69] for the prediction of stable vs. progressive mild cognitive impairment (MCI) conversion in Alzheimer’s disease with multi-task learning and Convolutional Neural Networks [70]. PDE-LDDMM overpassed ANTS-lNCC for this task, in terms of accuracy, sensitivity, and specificity. ANTS-lNCC obtained a median accuracy value of 84%, a sensitivity of 88% and specificity of 81%. Variant II with NCC achieved the best performing accuracy, with a median value of 89%, and sensitivity and specificity values among the best ones, with a median value of 94% and 91%, respectively. Indeed, NCC overpassed lNCC metric in this task, despite the comparable performance achieved by both metrics in the template-based evaluation presented in this work. As future work, we will perform a comprehensive study to find out the whys behind the improved performance of a given configuration with respect to the others.

Our PDE-LDDMM method may serve as a benchmark method for the exploration of different image similarity metrics in the loss function of deep-learning methods. In addition, it may be a good candidate in applications where there are not enough data to generate accurate learning-based models. Even more, it may be used as the backbone of hybrid approaches that combine traditional with modern learning-based models which are being pointed out as one promising research direction [55].

## Figures and Tables

**Table 1:** NIREP16. Multi-resolution experiments and Gauss–Newton–Krylov optimization. Results of ANOVA tests for the effects of method and image similarity metric selection.

Factor I	Factor II	*p*-Value I	*p*-Value II
Baseline vs. SP Variant I	$E_{img}$	$1.07\times 10^{-5}$	$1.09\times 10^{-11}$
Baseline vs. BL Variant I	$E_{img}$	$3.09\times 10^{-7}$	$1.45\times 10^{-19}$
Baseline vs. SP Variant II	$E_{img}$	$1.07\times 10^{-5}$	$1.09\times 10^{-11}$
Baseline vs. BL Variant II	$E_{img}$	$1.07\times 10^{-5}$	$1.09\times 10^{-11}$
PDE-LDDMM methods	$E_{img}$	$3.89\times 10^{-7}$	$1.77\times 10^{-21}$
SP Variant I vs. SP Variant II	$E_{img}$	0.339	0
BL Variant I vs. BL Variant II	$E_{img}$	$1.88\times 10^{-8}$	$3.46\times 10^{-13}$
SP Variant I vs. BL Variant I	$E_{img}$	0.0284	0
SP Variant II vs. BL Variant II	$E_{img}$	0.0068	0

**Table 2:** NIREP16. Convergence results. Mean and standard deviation of the relative image similarity error expressed in % ($MSE_{rel}$), the relative gradient magnitude (${∥g∥}_{\infty ,rel}$), and the Jacobian determinant extrema associated with the transformation ${\left(\phi_{1}^{v}\right)}^{-1}$. The best $MSE_{rel}$ results for each variant are highlighted in bold face.

Metric	${MSE}_{rel}\left(\%\right)$	${∥g∥}_{\infty ,rel}$	$\min(J{\left(\phi_{1}^{v}\right)}^{-1})$	$\max(J{\left(\phi_{1}^{v}\right)}^{-1})$
**Single-resolution**				
SP Variant I, SSD, GN	18.29 ± 2.83	0.07 ± 0.05	0.16 ± 0.05	3.70 ± 0.51
SP Variant I, NCC, GN	**16.51 ± 2.13**	0.17 ± 0.01	0.01 ± 0.02	3.92 ± 0.68
SP Variant I, lNCC, GN	20.56 ± 2.31	0.49 ± 0.32	0.04 ± 0.03	3.79 ± 0.40
SP Variant I, NGFs, GN	39.14 ± 2.91	0.71 ± 0.15	0.14 ± 0.03	4.67 ± 0.35
SP Variant I, MI, GD	24.34 ± 2.58	0.13 ± 0.06	0.18 ± 0.06	2.17 ± 0.15
SP Variant II, SSD, GN	17.10 ± 1.50	0.12 ± 0.05	0.14 ± 0.05	5.02 ± 1.01
SP Variant II, NCC, GN	**15.34 ± 1.95**	0.15 ± 0.06	0.10 ± 0.05	6.31 ± 1.65
SP Variant II, lNCC, GN	24.34 ± 2.90	0.30 ± 0.08	0.20 ± 0.03	3.71 ± 0.64
SP Variant II, NGFs, GN	46.78 ± 2.50	0.67 ± 0.13	0.34 ± 0.03	3.65 ± 0.46
SP Variant II, MI, GD	22.26 ± 2.42	0.12 ± 0.02	0.26 ± 0.05	2.61 ± 0.32
BL Variant I, SSD, GN	19.89 ± 1.76	0.01 ± 0.00	0.29 ± 0.03	3.45 ± 0.29
BL Variant I, NCC, GN	**17.06 ± 1.71**	0.03 ± 0.01	0.09 ± 0.04	4.56 ± 0.59
BL Variant I, lNCC, GN	24.30 ± 2.68	0.14 ± 0.06	0.17 ± 0.03	3.69 ± 0.49
BL Variant I, NGFs, GN	71.93 ± 1.78	0.78 ± 0.08	0.62 ± 0.02	1.56 ± 0.05
BL Variant I, MI, GD	26.21 ± 2.66	0.02 ± 0.00	0.28 ± 0.04	2.19 ± 0.14
BL Variant II, SSD, GN	17.77 ± 1.66	0.04 ± 0.01	0.13 ± 0.04	4.81 ± 0.72
BL Variant II, NCC, GN	**15.57 ± 1.71**	0.05 ± 0.02	0.09 ± 0.04	6.29 ± 1.15
BL Variant II, lNCC, GN	22.74 ± 2.77	0.16 ± 0.04	0.17 ± 0.04	4.41 ± 0.76
BL Variant II, NGFs, GN	71.77 ± 1.75	0.91 ± 0.08	0.60 ± 0.02	1.56 ± 0.05
BL Variant II, MI, GD	23.30 ± 2.61	0.03 ± 0.01	0.28 ± 0.04	2.46 ± 0.18
**Multi-resolution**				
SP Variant I, SSD, GN	17.98 ± 2.71	2.50 ± 3.49	0.02 ± 0.08	3.67 ± 0.82
SP Variant I, NCC, GN	14.89 ± 2.24	1.33 ± 0.89	0.04 ± 0.12	9.77 ± 20.45
SP Variant I, lNCC, GN	**12.94 ± 3.11**	0.72 ± 0.37	0.01 ± 0.02	5.17 ± 1.18
SP Variant I, NGFs, GN	29.08 ± 2.55	0.66 ± 0.26	0.05 ± 0.04	6.99 ± 1.74
SP Variant I, MI, GD	18.35 ± 2.19	0.99 ± 1.39	0.06 ± 0.06	3.26 ± 0.31
SP Variant II, SSD, GN	15.93 ± 1.51	1.13 ± 0.67	0.12 ± 0.03	9.89 ± 6.86
SP Variant II, NCC, GN	**13.12 ± 1.69**	1.02 ± 0.27	0.08 ± 0.03	14.32 ± 11.37
SP Variant II, lNCC, GN	15.23 ± 2.20	1.10 ± 0.39	0.10 ± 0.03	5.52 ± 0.81
SP Variant II, NGFs, GN	24.20 ± 2.41	0.84 ± 0.15	0.23 ± 0.03	4.76 ± 1.08
SP Variant II, MI, GD	19.15 ± 2.41	0.46 ± 0.08	0.17 ± 0.04	4.14 ± 1.30
BL Variant I, SSD, GN	19.22 ± 1.72	0.11 ± 0.05	0.14 ± 0.06	3.74 ± 0.38
BL Variant I, NCC, GN	15.81 ± 1.62	0.21 ± 0.07	0.09 ± 0.05	5.04 ± 0.76
BL Variant I, lNCC, GN	**14.41 ± 2.45**	0.82 ± 0.25	0.08 ± 0.04	6.32 ± 1.64
BL Variant I, NGFs, GN	24.10 ± 2.96	0.30 ± 0.15	0.09 ± 0.02	9.62 ± 1.90
BL Variant I, MI, GD	18.35 ± 2.19	0.31 ± 0.21	0.06 ± 0.06	3.26 ± 0.31
BL Variant II, SSD, GN	16.30 ± 1.60	0.25 ± 0.08	0.11 ± 0.04	6.30 ± 1.64
BL Variant II, NCC, GN	**13.65 ± 1.72**	0.47 ± 0.19	0.07 ± 0.03	9.46 ± 2.78
BL Variant II, lNCC, GN	13.67 ± 2.26	1.00 ± 0.48	0.08 ± 0.03	7.92 ± 1.23
BL Variant II, NGFs, GN	23.04 ± 2.25	0.51 ± 0.20	0.07 ± 0.02	7.72 ± 2.24
BL Variant II, MI, GD	19.35 ± 2.50	0.13 ± 0.15	0.17 ± 0.05	3.80 ± 0.63

**Table 3:** NIREP16. Quantitative results of the deep-learning methods. Mean and standard deviation of the relative image similarity error expressed in % ($MSE_{rel}$), and the Jacobian determinant extrema associated with the transformation $\phi^{-1}$. The best $MSE_{rel}$ results for each method are highlighted in bold face.

Metric	${MSE}_{rel}\left(\%\right)$	$\min(J\left(\phi^{-1}\right))$	$\max(J\left(\phi^{-1}\right))$
VM-SSD	17.82 ± 0.98	−13.04 ± 5.72	86.12 ± 97.05
VM-CC	20.25 ± 0.93	−14.89 ± 3.87	59.69 ± 11.59
VM2-SSD	16.35 ± 0.67	−9.26 ± 5.26	53.13 ± 26.16
VM2-CC	**14.34 ± 0.95**	−7.65 ± 1.56	46.91 ± 7.83
QuickSilver	19.03 ± 1.37	0.31 ± 0.03	9.54 ± 2.44
QuickSilver-C	**15.75 ± 1.27**	0.32 ± 0.05	9.59 ± 2.66

**Table 4:** NIREP16. Single-resolution experiments. Mean and standard deviation of the total GPU time, and maximum VRAM memory usage achieved by the methods through the registrations.

Method	${time}_{GPU}$ (s)	Peak VRAM (MBs)
SP Variant I, SSD, GN	144.51 ± 5.87	5823
SP Variant I, NCC, GN	145.25 ± 6.93	5875
SP Variant I, lNCC, GN	169.02 ± 0.92	6159
SP Variant I, NGFs, GD	149.18 ± 1.04	5023
SP Variant I, MI, GD	347.35 ± 0.41	4997
BL Variant I, SSD, GN	77.44 ± 0.28	3263
BL Variant I, NCC, GN	79.92 ± 0.51	3279
BL Variant I, lNCC, GN	109.69 ± 1.45	3705
BL Variant I, NGFs, GD	132.41 ± 1.71	2945
BL Variant I, MI, GD	328.27 ± 0.47	2977
SP Variant II, SSD, GN	215.61 ± 1.66	5769
SP Variant II, NCC, GN	216.29 ± 3.25	5899
SP Variant II, lNCC, GN	238.49 ± 3.25	6065
SP Variant II, NGFs, GD	159.21 ± 1.58	4555
SP Variant II, MI, GD	354.55 ± 2.11	4555
BL Variant II, SSD, GN	100.07 ± 0.30	2271
BL Variant II, NCC, GN	100.21 ± 0.88	2557
BL Variant II, lNCC, GN	130.53 ± 1.01	2921
BL Variant II, NGFs, GD	138.28 ± 1.87	2389
BL Variant II, MI, GD	331.28 ± 1.87	2357

**Figure A1:**
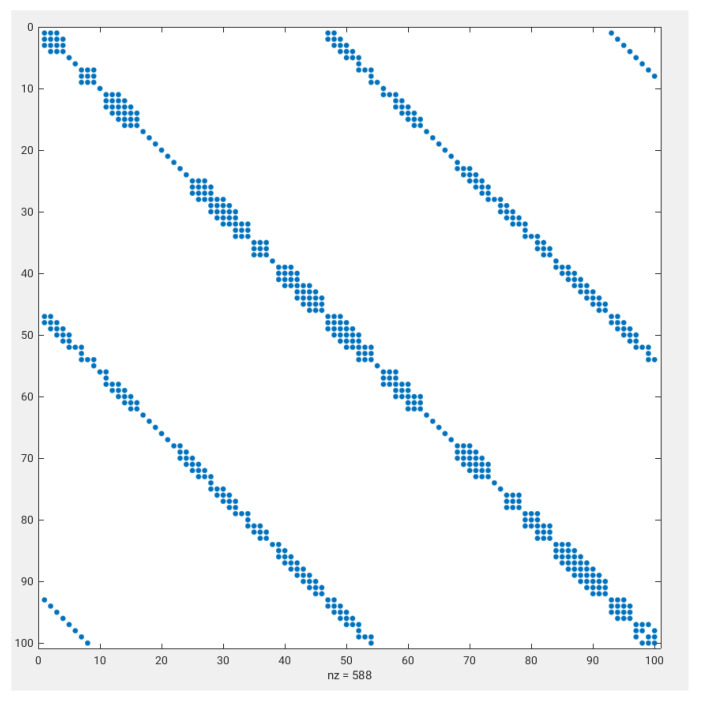
Block-diagonal pattern of the first 100×100 elements of the matrix $\left(\Lambda^{T}\cdot 2Id\cdot \Lambda \right)$.

**Figure A2:**
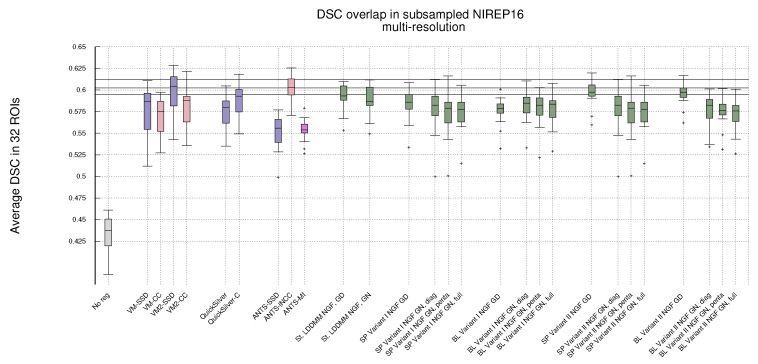
NIREP16. Volume overlap obtained by the NGFs PDE-LDDMM methods considered in this work. Methods differ on the approximation used for matrix $\left(\Lambda^{T}\cdot 2Id\cdot \Lambda \right)$: diagonal, penta-diagonal, and full-diagonal.

## Data Availability

Not applicable.

## Appendix A. Stationary LDDMM

Stationary LDDMM was proposed in [67,71] as a lightweight approach to the original LDDMM problem proposed in [56]. The method replaces the non-stationary parameterization of diffeomorphisms by steady velocity fields and it uses Gauss–Newton optimization with a block-diagonal approximation of the Hessian matrix. The result is a method close to the LDDMM foundations and competitive with Diffeomorphic Demons [71,72].

In a nutshell, St-LDDMM is formulated from the minimization of the energy functional

(A1) $$E\left(v\right)=\frac{1}{2}{∥v∥}_{V}^{2}+\frac{1}{\sigma^{2}}{∥I_{0}\circ \varphi^{-1}-I_{1}∥}_{L^{2}}^{2},$$

where v∈V is an steady velocity field and φ is the solution of the transport equation

(A2) $$\frac{d\varphi }{dt}=-v\circ \varphi ,$$

that is solved using scaling and squaring [73].

The gradient can be obtained from the first-order G$\hat{a}$teaux derivative of E(v) and the identity

(A3) $$\partial_{\eta }E\left(v\right)=<\nabla_{v}E\left(v\right),\eta {>}_{V}.$$

Thus, for the SSD image similarity metric,

(A4) $$\nabla_{v}E\left(v\right)=v-\frac{2}{\sigma^{2}}K\left(\left(I_{0}\circ \varphi^{-1}-I_{1}\right)D\left(I_{0}\circ \varphi^{-1}\right)\right).$$

The energy gradient can be similarly derived for the different $E_{\mathrm{img}}$ metrics considered in this work yielding

(A5) $$\nabla_{v}E\left(v\right)=v-\frac{2}{\sigma^{2}}K\left(\alpha_{g}D\left(I_{0}\circ \varphi^{-1}\right)\right),$$

where $\alpha_{g}$ is an scalar field corresponding with the differential of the metric with respect to the residual $I_{0}\circ \varphi^{-1}-I_{1}$. Table A1 gathers the expressions of $\alpha_{g}$, which can be easily identified from the derivations of the PDE-LDDMM methods proposed in this work.

The Hessian can be obtained from the second-order G$\hat{a}$teaux derivative of E(v) and the identity

(A6) $$\partial_{\eta \eta }E\left(v\right)=<\eta ,H_{v}E\left(v\right)\eta {>}_{V}.$$

For Gauss–Newton optimization, the Hessian is approximated by a block-diagonal positive-definite matrix yielding

(A7) $$H_{v}E\left(v\right)\approx Id_{d}+\frac{2}{\sigma^{2}}K\left(D(I_{0}\circ \varphi^{-1})\right)\cdot K\left(D(I_{0}\circ \varphi^{-1})\right).$$

for the SSD metric. For the other $E_{img}$ metrics, the energy Hessian is given by

(A8) $$H_{v}E\left(v\right)\approx Id_{d}-\frac{2}{\sigma^{2}}K\left(\alpha_{h}D\left(I_{0}\circ \varphi^{-1}\right)\right)\cdot K\left(D(I_{0}\circ \varphi^{-1})\right),$$

where $\alpha_{h}$ is a scalar field corresponding with the differential of the scalar field $\alpha_{g}$. Table A1 gathers the expressions of $\alpha_{h}$.

St-LDDMM has been included in our comparison in order to demonstrate the superiority of PDE-LDDMM over unconstrained approaches and the correctness of the derivations of $\alpha_{g}$ and $\alpha_{h}$ for NGFs and Gauss–Newton optimization.

**Table A1:** Scalar fields from the gradients and Hessians of St-LDDMM with the different $E_{img}$ metrics considered in this work. The values of *A*, *B*, *C*, and Θ are analogs of the ones derived in the main manuscript for PDE-LDDMM replacing m(1) with $I_{0}\circ \varphi^{-1}$. The notation ${\left(\cdot \right)}_{\nu }$ in lNCC indicates the restriction of the computation to a neighborhood of size ν.

Metric	$\alpha_{g}$	$\alpha_{h}$
SSD	$\left(I_{0}\circ \varphi^{-1}-I_{1}\right)$	Id
NCC	$\frac{2}{C}\left(\frac{A}{B}{\bar{I}}_{1}-{\left(\frac{A}{B}\right)}^{2}\bar{I_{0}\circ \varphi^{-1}}\right)$	$\frac{2}{C}\left(\Theta {\bar{I}}_{1}-2\frac{A}{B}\Theta \bar{I_{0}\circ \varphi^{-1}}-{\left(\frac{A}{B}\right)}^{2}\right)$
lNCC	${\left(\frac{2}{C}\left(\frac{A}{B}{\bar{I}}_{1}-{\left(\frac{A}{B}\right)}^{2}\bar{I_{0}\circ \varphi^{-1}}\right)\right)}_{\nu }$	${\left(\frac{2}{C}\left(\Theta {\bar{I}}_{1}-2\frac{A}{B}\Theta \bar{I_{0}\circ \varphi^{-1}}-{\left(\frac{A}{B}\right)}^{2}\right)\right)}_{\nu }$
NGFs	$-2\nabla \cdot \left(\frac{A}{{\left(BC\right)}^{2}}\nabla I_{1}-{\left(\frac{A}{B^{2}C}\right)}^{2}\nabla \left(I_{0}\circ \varphi^{-1}\right)\right)$	$2\alpha^{T}\cdot \alpha$
MI	$-\sum_{r,s}\frac{1}{N_{x}}\sum_{x}\frac{\partial \xi }{\partial r}\xi \left(s-{\bar{I}}_{1}\left(x\right)\right)\left(1+\log\frac{p_{I_{0}\circ \varphi^{-1},I_{1}}\left(r,s\right)}{p_{I_{0}\circ \varphi^{-1}}\left(r\right)}\right)$	n.a.

## Appendix B. Remarks on Normalized Gradient Fields (NGFs) Metric

Unlike NCC and lNCC metrics, the differentiation of λ for NGFs needed in Gauss–Newton–Krylov optimization yields a complex differential expression for δλ(1). In addition, the computation of δλ(1) in differential form may yield numerical problems during the optimization. For these reasons, the differentiation of λ for the computation of δλ(1) has been approached in this work writing the differential operators in matrix form after problem discretization, following the ideas in [32].

Thus, let $G_{x}$, $G_{y}$, and $G_{z}$ be the matrices associated with the discretization of the partial differential operators $\partial_{x}$, $\partial_{y}$, and $\partial_{z}$. Recall the expression of λ(1) in Equation (59)

(A9) $$\begin{array}{c} \lambda \left(1\right)=-\frac{2}{\sigma^{2}}\nabla \cdot \left(\frac{A}{{\left(BC\right)}^{2}}\nabla I_{1}-{\left(\frac{A}{B^{2}C}\right)}^{2}\nabla m\left(1\right)\right). \end{array}$$

Using the matrix form of the gradient *∇* and the divergence ∇· operators, λ(1) can be written as

(A10) $$\begin{array}{cc} \lambda (1)= & \left(\frac{A}{{\left(BC\right)}^{2}}I_{1}⊙Gx-{\left(\frac{A}{B^{2}C}\right)}^{2}m\left(1\right)⊙Gx\right)⊙Gx\\ & +\left(\frac{A}{{\left(BC\right)}^{2}}I_{1}⊙Gy-{\left(\frac{A}{B^{2}C}\right)}^{2}m\left(1\right)⊙Gy\right)⊙Gy\\ & +\left(\frac{A}{{\left(BC\right)}^{2}}I_{1}⊙Gz-{\left(\frac{A}{B^{2}C}\right)}^{2}m\left(1\right)⊙Gz\right)⊙Gz \end{array}$$

where ⊙ represents the Hadamard matrix product. Then, denoting by Λ the matrix representation of λ(1), the Gauss–Newton approximation of δλ(1) can be computed from

(A11) $$\delta \lambda \left(1\right)=dm{\left(1\right)}^{T}\cdot \left(\Lambda^{T}\cdot 2Id\cdot \Lambda \right).$$

Figure A1 shows the block-diagonal pattern of the matrix $\Lambda^{T}\cdot 2Id\cdot \Lambda$, involved in the computation of δλ(1). The matrix is a band matrix with twenty-five not-null diagonals. The distribution of not-null diagonals involves one central pentadiagonal structure, two symmetrically located tridiagonal structures, and the remaining symmetrically located diagonals up to completing the twenty-five diagonals of the band structure. The complexity of the diagonal pattern gives an idea of the complexity of the stencil associated with the differential expression for δλ(1).

Inspired on the simpler block-diagonal structure of the Gauss–Newton version of St-LDDMM proposed in [67], we have compared the performance of NGFs PDE-LDDMM using diagonal, penta-diagonal, and all-diagonal truncations of $\left(\Lambda^{T}\cdot 2Id\cdot \Lambda \right)$. Figure A2 shows the DSC metrics obtained by the different methods in NIREP16. The figure shows that the diagonal approximation slightly outperforms the penta- and full-diagonal approximations for both Variant I and II. The performance of the method with the diagonal approximation shows an identical distribution between the spatial variants and approximately identical between the BL variants. For Variant I, the performance of Gauss–Newton–Krylov optimization is similar to gradient-descent optimization. However, for Variant II, the performance of gradient-descent optimization is strikingly superior.

Table A2 shows the mean and the standard deviation of $MSE_{rel}$, ${∥g∥}_{\infty ,rel}$, and the extrema of the Jacobian determinant obtained with the methods. For the spatial methods, gradient descent obtains the lowest $MSE_{rel}$ values. For the BL methods, Gauss–Newton with the diagonal truncation shares the lowest $MSE_{rel}$ values with gradient descent. The Jacobians reach more extreme values for the Gauss–Newton methods. These results break with the trend that we have observed for SSD and NCC-like metrics and open the possibility that, in some cases, gradient-descent finds better performing minima than Gauss–Newton–Krylov and it is, therefore, a preferable option.

**Table A2:** NIREP16. Convergence results. Mean and standard deviation of the relative image similarity error expressed in % ($MSE_{rel}$), the relative gradient magnitude (${∥g∥}_{\infty ,rel}$), and the Jacobian determinant extrema associated with the transformation ${\left(\phi_{1}^{v}\right)}^{-1}$.

Multi-Resolution				
Metric	${MSE}_{rel}\left(\%\right)$	${∥g∥}_{\infty ,rel}$	$\min(J{\left(\phi_{1}^{v}\right)}^{-1})$	$\max(J{\left(\phi_{1}^{v}\right)}^{-1})$
SP Variant I, NGFs, GD	22.98 ± 2.54	0.56 ± 0.66	0.14 ± 0.05	4.57 ± 0.46
SP Variant I, NGFs, GN, FD	29.08 ± 2.55	0.66 ± 0.26	0.05 ± 0.04	6.99 ± 1.74
SP Variant I, NGFs, GN, PD	28.86 ± 3.32	0.64 ± 0.17	0.04 ± 0.02	8.95 ± 2.32
SP Variant I, NGFs, GN, D	27.22 ± 2.31	0.68 ± 0.24	0.03 ± 0.02	8.96 ± 1.75
SP Variant II, NGFs, GD	20.63 ± 2.78	0.57 ± 0.28	0.23 ± 0.05	6.88 ± 1.59
SP Variant II, NGFs, GN, FD	24.20 ± 2.41	0.84 ± 0.15	0.23 ± 0.03	4.76 ± 1.08
SP Variant II, NGFs, GN, PD	23.92 ± 3.02	0.94 ± 0.08	0.20 ± 0.03	7.04 ± 2.20
SP Variant II, NGFs, GN, D	23.42 ± 2.74	0.85 ± 0.16	0.19 ± 0.03	7.38 ± 2.40
BL Variant I, NGFs, GD	24.38 ± 2.65	0.08 ± 0.02	0.21 ± 0.06	5.41 ± 0.85
BL Variant I, NGFs, GN, FD	24.10 ± 2.96	0.30 ± 0.15	0.09 ± 0.02	9.62 ± 1.90
BL Variant I, NGFs, GN, PD	23.45 ± 2.47	0.33 ± 0.19	0.09 ± 0.02	12.93 ± 3.35
BL Variant I, NGFs, GN, D	23.31 ± 2.16	0.37 ± 0.23	0.09 ± 0.02	12.07 ± 3.16
BL Variant II, NGFs, GD	21.32 ± 2.80	0.09 ± 0.05	0.22 ± 0.05	5.82 ± 0.96
BL Variant II, NGFs, GN, FD	23.04 ± 2.25	0.51 ± 0.20	0.07 ± 0.02	7.72 ± 2.24
BL Variant II, NGFs, GN, PD	22.39 ± 2.28	0.47 ± 0.23	0.08 ± 0.02	7.66 ± 1.49
BL Variant II, NGFs, GN, D	21.98 ± 2.14	0.53 ± 0.32	0.07 ± 0.02	8.16 ± 1.50
